# Spatial modeling of forest-savanna bistability: impacts of fire dynamics and timescale separation

**DOI:** 10.1007/s00285-026-02363-9

**Published:** 2026-03-05

**Authors:** Kimberly Shen, Simon Levin, Denis Patterson

**Affiliations:** 1 Department of Physics, Princeton University, Princeton, NJ 08544 USA; 2 Peabody Institute, Johns Hopkins University, Baltimore, MD 21218 USA; 3 Department of Mathematical Sciences, Durham University, Stockton Road, Durham DH1 3LE, UK; 4 Department of Ecology and Evolutionary Biology, Princeton University, Princeton, NJ 08544 USA; 5 High Meadows Environmental Institute, Princeton University, Princeton, NJ 08544 USA

**Keywords:** Spatial stochastic processes, Ecology, Mean-field models, Bifurcation analysis, Partial integro-differential equations, Tree-grass coexistence, 65C40, 34C23, 92B05

## Abstract

Forest-savanna bistability – the hypothesis that forests and savannas exist as alternative stable states in the tropics – and its implications are key challenges for mathematical modelers and ecologists in the context of ongoing climate change. To generate new insights into this problem, we present a spatial Markov jump process model of savanna forest fires that integrates key ecological processes, including seed dispersal, fire spread, and non-linear vegetation flammability. In contrast to many models of forest-savanna bistability, we explicitly model both fire dynamics and vegetation regrowth in a mathematically tractable framework. This approach bridges the gap between slow-timescale vegetation models and highly resolved fire dynamics, shedding light on the influence of short-term and transient processes on vegetation cover. In our spatial stochastic model, bistability arises from periodic fires that maintain low forest cover, whereas dense forest areas inhibit fire spread and preserve high tree density. The deterministic mean-field approximation of the model similarly predicts bistability, but deviates quantitatively from the fully spatial model, especially in terms of its transient dynamics. These results also underscore the critical role of timescale separation between fire and vegetation processes in shaping ecosystem structure and resilience.

## Introduction

Satellite data of tree cover in Sub-Saharan Africa combined with mean annual rainfall (MAR) records have shown that tree cover exhibits a distinctly bimodal distribution in regions with intermediate MAR (1000 to 2000 mm/yr). Savannas (regions with sparse open-canopy tree cover less than $\approx 40 \%$ per unit area) and forests (regions with high closed-canopy tree cover above $\approx 70 \%$ per unit area) are frequently observed in the tropics, while intermediate tree cover (40 to $70 \%$) is rarely observed (Van Nes et al. 2018). The notion of forests and savannas existing as alternative stable states has attracted a significant amount of attention and concern since it suggests that perturbations caused by climate change, drought, or human activity can cause large-scale conversion of forests to savanna, which may be extremely difficult to reverse due to hysteresis effects (Cochrane et al. 1999).

The preservation of forests, in turn, is a crucial concern in mitigating climate change, as forests have significantly higher carbon storage capacities than savannas. Forests also support local human populations by providing building materials. In addition to forests, the maintenance of savannas is also critical to biodiversity and conservation efforts due to the high species richness of ecosystems located at the savanna-forest ecotone (Behling et al. 2007; Myers et al. 2000; Overbeck et al. 2007). Therefore, a model that accurately predicts the responses of forest-savanna mosaics to various environmental disturbances would usefully inform various climate- and biodiversity-related interventions, such as selecting and maintaining protected areas and regulations of human ecological disturbances. As such, a maximally valuable model must encompass short-term disturbances, such as fires, and long-term effects, such as forest encroachment or loss. The model ideally must also be spatially explicit since the fundamental driving processes of the system (e.g., seed dispersal and fire spread) have a highly spatially inhomogeneous structure.

A widely accepted explanation for the observed bimodal distribution of forests and savannas is that regular disturbance by fires can maintain open-canopy savannas. In contrast, denser closed-canopy forests sufficiently suppress the flammable grassy layer, which limits fire spread and lowers the probability of large fires, stabilizing the forest state. Mechanistically, fires typically spread readily within savannas fueled by highly flammable grasses but do not significantly penetrate forest boundaries (Biddulph and Kellman 1998; Hennenberg et al. 2008; Kellman and Meave 1997; Bond 2008; Hoffmann et al. 2009; Swaine et al. 1992). Thus, regions with sufficiently high grass cover may burn frequently enough to maintain savanna conditions despite climatological conditions supporting forest growth (Brando et al. 2012, 2014; Cochrane and Laurance 2002). The original Staver-Levin model (Staver et al. 2011; Staver and Levin 2012) and related models (Accatino et al. 2010; Tilman 1994), which incorporate fire-vegetation interactions in a spatially implicit mean-field approximation, have been widely used to explain and study forest-savanna bistability (Staal et al. 2018; Nes et al. 2014; Yatat et al. 2018; Wuyts et al. 2017). We will use an extension of the Staver-Levin modeling framework as the basis for our study, but our conclusions apply more broadly across this class of spatially implicit forest-savanna models.

Many forest fire models, such as the Staver-Levin model, use mean-field approximations to remain interpretable and analytically tractable, while only indirectly incorporating spatial processes. For example, Klimasara and Tyran-Kamińska (2023); Magnani et al. (2023); Hoyer-Leitzel and Iams (2021) are recently proposed models that use a mean-field (i.e., spatially implicit) approach but add discontinuous, stochastic changes in vegetation proportions to implicitly account for fire spreading events. However, explicitly spatial models have some advantages over non-spatial models, which implicitly incorporate spatial effects (DeAngelis and Yurek 2017). For instance, spatial models have greater practical applicability and more accurately replicate the behavior of systems in which local mechanisms amplified by positive feedback, such as fire, can cause large-scale effects. Thus, to construct an accurate and practically applicable model, it is necessary to include sufficient detail to model both local conditions and the dynamics that they generate. Spatial structure can also profoundly affect the behavior of a system. For instance, Durrett and Ma (2018) found that in a spatial model of grass-forest competition, both states can co-exist only when transition rates are spatially heterogeneous.

Various other spatial models of fires in savanna-forest systems have already been proposed and studied, but have shortcomings. For example, some forest fire models are parametrized based on empirical data (Lasslop et al. 2016; Archibald et al. 2012; Aleman and Staver 2018; Rammer and Seidl 2019), but such models cannot transparently demonstrate the system’s underlying dynamics nor be applied to novel situations. Other studies based on field sampling of species diversity, fire frequency, and/or soil quality have similar drawbacks (Beckett et al. 2022; Sagang et al. 2024). Some mean-field models have been extended to incorporate spatial processes (seed dispersal, fire) by adding diffusion terms to the nonspatial dynamics (Zelnik et al. 2024; Goel et al. 2020), but these models do not explicitly model fire dynamics. Explicit fire dynamics are essential for examining the behavior of grass-forest systems on shorter timescales and for studying the effects of seasonally dependent flammability on forest dynamics (Scholes and Archer 1997). Nonetheless, these studies have already produced interesting results, such as the dependence of forest front travel speed on the curvature of the front and the need for a critical patch size to ensure growth, adding a hysteresis-like quality to the system’s behavior (Durrett and Ma 2018; Goel et al. 2020).

Most spatially explicit forest fire models employ a square grid and use nearest neighbor-dependent transition probabilities to model spatial processes such as fire spread and seed dispersal (Wuyts and Sieber 2023; Staal et al. 2018; Van Nes et al. 2018; Hochberg et al. 1994; Accatino et al. 2016; Li et al. 2019; Abades et al. 2014; Fair et al. 2020). These models have already led to notable findings. For example, Wuyts and Sieber (2023) explicitly demonstrated that fire-vegetation feedback can maintain bistability in tree cover states, Fair et al. (2020) found that forest-savanna mosaics occur under limited conditions, suggesting that such mosaics are vulnerable to loss under climate change, and Abades et al. (2014) found a second order phase transition in spanning cluster formation probability. However, these models have some intrinsic limitations. For instance, they cannot incorporate inhomogeneities in vegetation density and long-distance or anisotropic spreading of fire and seeds. Additionally, the ability of these models to explain observations on short time horizons may be limited by the relatively short timescale of fire dynamics compared to the timestep increment (typically 1 year).

The paper is organized as follows: Section 2 introduces the spatial stochastic modeling framework that forms the basis for this study, including a discussion on plausible parameter regimes and rigorous links to continuum models. Section 3 provides a bifurcation analysis of a nonspatial version of the model for later comparison with the spatial version and other similar nonspatial forest-savanna models in the extant literature. Section 4 studies the dynamics of the full spatial stochastic model and contrasts them with the dynamics of the nonspatial model from the previous section, emphasizing the key role of transient dynamics on short timescales. Finally, Section 5 concludes with a synthesis of the main findings and highlights some future directions and open problems in the mathematical modeling of forest-savanna bistability.

## A Spatial Stochastic Forest Fire Model

Ecologically, tropical forests differ markedly from coniferous forests in several key ways. First, savanna trees are not readily killed by fire. Typically, they are only top-killed (i.e., only aerial biomass is burned) and can readily resprout (Hoffmann et al. 2003, 2009; Balch et al. 2011) or have thickened bark to prevent stem death (Hoffmann et al. 2012). Second, fire does not propagate readily through tropical forests (Hennenberg et al. 2006; Archibald et al. 2009; Pueyo et al. 2010) but does propagate rapidly through savanna grassland (Wragg et al. 2018). Mechanistically, this is due to forest understory shade excluding flammable C4 grasses (Hoffmann et al. 2012) in addition to reduced wind speeds and increased moisture in the forest microclimate (Hoffmann et al. 2012; Hennenberg et al. 2008). For brevity, we will henceforth refer to tropical forests as “forest” and savanna regions with lower tree density as “grassland”.

### Mathematical Description of the Model

We use a spatially extended Markov jump process to model the dynamics of fires in forest-grass systems using the mathematical framework introduced in Patterson et al. (2020). The set $\Omega \subset \mathbb {R}^2$ denotes the spatial domain and the random variables $\{r_i \in \Omega :\, i = 1,\dots , N \}$ are sites (locations) where vegetation is present or has the potential to grow. Each site *i*, with respective location $r_i$, undergoes a Markov jump process with possible states *F* (forest), *G* (grass), *B* (burning), and *A* (ash). We refer to models with this state space as the “FGBA model” for brevity. The “ash” state refers to areas of land that have recently burned and thus do not contain flammable cover or vegetation that can immediately burn (Hébert-Dufresne et al. 2018; Wuyts et al. 2017). We can thus consider ash to be a transitory or refractory state, and it is often omitted from models that only consider vegetation on longer timescales (years, decades). Still, it can play an important role in ecosystem dynamics on shorter timescales, which is the focus of the present work.

The transition rates between the FGBA model states depend on the spatial locations and states of all the other sites at locations $\{r_j \in \Omega \,: \, j = 1,\dots ,N, j \ne i\}$. This allows us to model both spatial spreading processes (e.g. forest and fire spreading) as well as non-spatial spontaneous transitions (e.g. fires ignited due to lightning strikes or human activity (Archibald et al. 2012)). The spatial locations of the vegetation can be drawn from any sufficiently regular distribution on $\Omega$, but, in this paper, we draw from a uniform distribution on $\Omega$ to reflect a homogeneous domain scenario. In the limit as $N \rightarrow \infty$, we completely fill the spatial domain with possible sites for vegetation. We can then obtain an approximate continuum model that can be studied as either a spatial mean-field model (via a system of integro-differential equations) or as a non-spatial mean-field model that presents as a system of ordinary differential equations (see Section 2.2 below for details).

We make the following assumptions about the possible transitions in our model:

1. Forest trees can expand into nearby grass and ash due to local spreading of seeds,
2. burning sites can expand into nearby forest and grass due to local fire spread,
3. Burning sites are spontaneously quenched into ash at a fixed rate,
4. Grass can regrow spontaneously from ash at a fixed rate due to homogeneous dispersal of grass seeds,
5. Forest can spontaneously transition to grass at a fixed rate due to non-fire related mortality.

Mathematically, we represent these assumptions by allowing each site to transition between the states *F*, *G*, *B*, *A* at exponentially distributed times with rates given by the sum of spatial spreading and spontaneous processes (see Table 1). The parameters in the transition rates are defined as follows:

- $\varphi _G, \varphi _A$ are constants controlling the rate of forest seeding into grass and into ash, respectively.
- $\beta _F, \beta _G$ are constants controlling the rate of fire spread within forest and within grass, respectively.
- $W_F, W_B: \Omega \times \Omega \rightarrow \mathbb {R}^+$ are forest spread and burning spread kernels, respectively, which control the extent of spatial spreading as a function of distance between the interacting sites. We assume that the kernels are $\mathcal {C}^{\infty }$.

**Table 1:** Transition rates between states in the spatial-stochastic FGBA model and their corresponding ecological processes

Transition	Transition rate at site *i*	Ecological process
$G \rightarrow F$	$\frac{ \varphi _G}{N} \sum _{j=1}^N W_F(r_i,\,r_j) 1\!\!1_{\{X^j(t)=F\}}$	forest spreading into grass
$A \rightarrow F$	$\frac{\varphi _A}{N} \sum _{j=1}^NW_F(r_i,\,r_j) 1\!\!1_{\{X^j(t)=F\}}$	forest spreading into ash
$F \rightarrow B$	$\frac{\beta _F}{N}\sum _{j=1}^N W_B(r_i,\,r_j)1\!\!1_{\{X^j(t)=B\}}$	fire spreading into forest
$G \rightarrow B$	$\frac{\beta _G}{N}\sum _{j=1}^NW_B(r_i,\,r_j)1\!\!1_{\{X^j(t)=B\}}$	fire spreading into grass
$F \rightarrow G$	$\mu$	non-fire forest mortality
$B \rightarrow A$	*q*	fire quenching
$A \rightarrow G$	$\gamma$	grass regrowth from ash

For the neighbor (local) transition rate parameters for fire and forest invasion ($\varphi _G$, $\varphi _A$, $\beta _F$, $\beta _G$), the subscript is the first English letter of the state before the relevant transition and the main letter is the Greek letter of the state after the relevant transition. We use Greek letters without subscripts for spontaneous transition rates ($\mu , q, \gamma$). We normalize the neighbor spread rates by $\frac{\text {area}(\Omega )}{N}$ to ensure that the spreading rates remain bounded in the limit $N \rightarrow \infty$.

In addition to the diffusive mode of fire spread described by the $W_B$ kernel and $\beta _G, \beta _F$ parameters, our modeling framework also includes a “cascade” mode of vegetation burning motivated by percolation theory. A key finding of detailed percolation-based fire models is that the probability of ignition for grass sites increases from a low baseline level to near one once the fraction of grass sites in the landscape exceeds a critical percolation threshold (with the exact value depending on the specific model) (Abades et al. 2014; Schertzer et al. 2015). This mechanism is also well supported by empirical data for forest-savanna ecosystems (Archibald et al. 2009; Pueyo et al. 2010; Van Nes et al. 2018). We incorporate this effect of a critical threshold for fire spread as a function of flammable cover by adding the terms

$$\begin{aligned} \Phi _G\bigg (\frac{1}{N}\sum _{i=1}^N W_G(r_i,\,r_j)1\!\!1_{\{X^j(t)=G\}}\bigg ) \quad \text { and } \quad \Phi _F\bigg (\frac{1}{N}\sum _{i=1}^N W_G(r_i,\,r_j)1\!\!1_{\{X^j(t)=G\}}\bigg ) \end{aligned}$$

to the $G \rightarrow B$ and $F \rightarrow B$ transition rates of site *i* at time *t*. Here $W_G: \Omega \times \Omega \rightarrow \mathbb {R}^+$ is another smooth spreading kernel, and $\Phi _G, \Phi _F:\mathbb {R}^+ \rightarrow \mathbb {R}^+$ are smooth sigmoidal functions which output flammability as a function of the local grass cover and the kernel $W_G$. More explicitly, we assume that $\Phi _G(\cdot )$ and $\Phi _F(\cdot )$ will take the form

$$\begin{aligned} \Phi _G(x) = g_0 + \frac{g_1 - g_0}{1 + e^{-(x-\theta _G)/s_G}} \quad \text { and } \quad \Phi _F(x) = f_0 + \frac{f_1 - f_0}{1 + e^{-(x-\theta _F)/s_F}} \end{aligned}$$

where the parameters are defined as follows:

- $f_0, g_0$ are the baseline spontaneous flammability of forest or grass sites when no other grass sites are present, e.g. ignitions due to lightning strikes or human activity (Archibald et al. 2012).
- $f_1, g_1$ are the total flammability of a grass or forest site, respectively in the limit when the land patch is purely grassland.
- $\theta _{F}, \theta _G$ are the percolation thresholds in forest and grass, respectively. We will use $\theta = \theta _F = \theta _G \approx 0.6$ as used for percolation in a square lattice (Gebele 1984).
- $s_F, s_G$ are non-negative constants controlling the width of forest and grass sigmoids, respectively. The sigmoids are assumed to be smooth approximations of increasing step functions, so we set $s_F = s_G = 0.05$.

The FGBA model outlined above spans multiple distinct timescales. Fire dynamics occur on a time scale of hours, grass regrowth on a time scale of months, and forest dynamics occur on a time scale of decades. In particular, we will estimate the parameter values from the expected time between events. Since we assume the transitions follow an exponential distribution, the rates are the inverse of the expected time between events. The rate parameters all have the same units of $\hbox {yr}^{-1}$, and we assume that the vegetation sites are separated by an average distance of 1 m.

The vegetation sites are chosen randomly within a compact square domain $[0, L] \times [0, L] \subset \mathbb {R}^2$ for some fixed $L >0$. We use periodic boundary conditions to reduce boundary effects and to model an infinite domain. The distance between two points $r, s \in [0, L] \times [0,L]$ is then computed as

$$\begin{aligned} |r - s|^2 = \frac{L}{2\pi } \Big ( \Big [ \text {arg}\Big (e^{i \frac{2 \pi (r_x - s_x)}{L}}\Big ) \Big ]^2 + \Big [ \text {arg}\Big (e^{i \frac{2 \pi (r_y - s_y)}{L}}\Big ) \Big ]^2 \Big ) \end{aligned}$$

where the argument function has range $[0, 2\pi )$.

We use Gaussian functions for the spreading kernels $W_G$, $W_F$ and $W_B$, although in principle, different functions could be used to model alternative spreading mechanisms if desired (such as very long-range seed dispersal modeled by heavy-tailed kernels). A vegetation site located at $r_i \in \Omega \subset \mathbb {R}^2$ has spreading kernels

$$\begin{aligned} W_{\mathcal {X}}(r_j, r_i)&= \frac{1}{2 \pi \sigma _{\mathcal {X}}^2}\text {exp}\Big (-\frac{|r_j - r_i|^2}{2\sigma _{\mathcal {X}}^2}\Big ) \end{aligned}$$

where $\mathcal {X} \in \{G,\, F,\,B\}$. To preserve the physical meaning of the three parameters $\sigma _{\mathcal {X}}$ under changes in *L* and/or *N*, we choose the values of $\sigma _{\mathcal {X}}$ to be in units of the average spacing between the vegetation sites in the domain which we define as $\Delta x \equiv \frac{L}{\sqrt{N}}$. The normalization constant of $W_{\mathcal {X}}$ is thus chosen such that

1 $$\begin{aligned} \lim _{N \rightarrow \infty } \frac{1}{N}\sum _{i=1}^NW_{\mathcal {X}}(r_j, r_i)&= \int _{\Omega } W_{\mathcal {X}}(r_j, r_i) \, dr_i = 1, \end{aligned}$$

We refer to the spatial Markov jump-process model outlined above as the **spatial-stochastic FGBA model** to avoid confusion with the mean-field versions (both spatial and non-spatial) that will also feature in our analysis and discussion. The model described in this section is similar to previous models proposed by Hébert-Dufresne et al. (2018) and Wuyts and Sieber (2023), particularly the choice of the state space, but has several distinguishing features. Firstly, our model allows the sites to be distributed arbitrarily in $\Omega$, while the related models use a square lattice of sites. Secondly, Hébert-Dufresne et al. (2018) and Wuyts and Sieber (2023) include additional spontaneous transitions $G, A, \rightarrow F$ to model the homogeneous long-distance dispersal of forest seeds. Our model more realistically incorporates long-distance forest seed dispersal by allowing an appropriately heavy-tailed kernel for $W_F$. Lastly, the related models assume that spreading processes occur strictly via nearest-neighbor spreading across adjacent lattice sites, while our model allows for more general spreading mechanisms via heavy-tailed kernels often used for seed dispersal (Nathan et al. 2012). Moreover, as we show below, our spatial stochastic model has immediate, rigorous connections with both spatial and non-spatial mean-field versions, which greatly assist with analysis and interpretability (see Section 2.2).

#### Parameter estimates

In this section, we set reasonable ranges and approximate values for all parameters in the FGBA model. We assume that *L* represents the physical side length of the area of land under study. Then, the average spacing between vegetation sites, assuming a uniform probability distribution, is $\Delta x \equiv \frac{L}{\sqrt{N}}$. We now estimate the parameter values by assuming $\Delta x = 1$ m to fix the spatial scale of the model and obtain plausible order-of-magnitude parameter estimates relative to this scale. Our results are not intended to be quantitative in nature, but rather we will illustrate dynamics possible over broad ranges of the plausible parameter space. When adjusted for average grid size differences, our parameter estimates are in line with those of related spatial stochastic forest-savanna models that used empirically validated parameters (Hébert-Dufresne et al. 2018; Wuyts and Sieber 2023). Our baseline parameter estimates are as follows:

- A square patch of grassland of side length $\Delta x$ surrounded by fire will burn after several minutes. Using Table 1 and Eq. (1), the burning rate can be approximated as $\beta _G$ then $\beta _G \approx 10^5$ $\hbox {yr}^{-1}$.
- A square patch of forest of side length $\Delta x$, which is surrounded by fire, will burn after about an hour. The burning rate can be approximated as $\beta _F$ so $\beta _F \approx 10^4$ $\hbox {yr}^{-1}$.
- A burning site is expected to burn for several hours before turning into ash, i.e. $q \approx 10^3$ $\hbox {yr}^{-1}$
- An ash site is expected to regrow grass in several months, i.e. $\gamma \approx 10^1$ $\hbox {yr}^{-1}$.
- A square patch of grass or ash of side length $\Delta x$ surrounded by forest will grow a forest tree after several years to a decade, i.e. $\varphi _A \approx \varphi _G \approx 10^{0}$ to $10^{-1}$ $\hbox {yr}^{-1}$.
- A tree at a square forest site of side length $\Delta x$ will spontaneously die from non-fire related causes after about 100 years, i.e. $\mu \approx 10^{-2}$ $\hbox {yr}^{-1}$.
- A square grass or forest vegetation site of side length $\Delta x$ completely surrounded by forest will spontaneously catch fire once every 100 years, i.e. $f_0, g_0 \approx 10^{-2}$.
- A grass or forest vegetation site will have increased flammability when completely surrounded by grass compared to a forest. We set $f_0 = 10^{-1}$ $\hbox {yr}^{-1}$ and $g_0 = 10^0$ $\hbox {yr}^{-1}$.
- The standard deviation of a fire site spreading throughout grass or forest vegetation is approximately 5 m, so set $\sigma _F \approx 5$ m.
- The standard deviation of a forest site spreading throughout grass or ash is approximately 5 m, so set $\sigma _B \approx 5$ m.
- The flammability of a vegetation site is dependent on grass proportions in a 5 m radius up to one standard deviation, so $\sigma _G \approx 5$ m.

We summarize the parameter values, and a representative fixed value is given below in Table 2. In this table, *G*(*t*) represents the fraction of the landscape occupied by grass sites.

**Table 2:** Estimates and representative values of the parameters for the FGBA model in units of $\hbox {yr}^{-1}$

Parameter	Value	Units	Ecological Interpretation
$\beta _G$	$10^5$	$\hbox {yr}^{-1}$	magnitude of fire spread rate over grass
$\beta _F$	$10^4$	$\hbox {yr}^{-1}$	magnitude of fire spread rate over forest
*q*	$10^4$	$\hbox {yr}^{-1}$	rate at which fire is quenched
$\gamma$	$10^2$	$\hbox {yr}^{-1}$	rate at which grass regrows from ash
$\varphi _A$	1	$\hbox {yr}^{-1}$	magnitude of forest spread rate over ash
$\varphi _G$	1	$\hbox {yr}^{-1}$	magnitude of forest spread rate over grass
$\mu$	$10^{-2}$	$\hbox {yr}^{-1}$	tree mortality rate due to non-fire-related causes
$f_0$	$10^{-2}$	$\hbox {yr}^{-1}$	spontaneous fire rate for each forest site when $G(t) \approx 0$
$f_1$	$10^{-1}$	$\hbox {yr}^{-1}$	spontaneous fire rate for each forest site when $G(t) \approx 1$
$g_0$	$10^{-2}$	$\hbox {yr}^{-1}$	spontaneous fire rate for each grass site when $G(t) \approx 0$
$g_1$	$10^{-1}$	$\hbox {yr}^{-1}$	spontaneous fire rate for each grass site when $G(t) \approx 1$
$\sigma _F$	5	m	width of forest spreading kernel
$\sigma _B$	5	m	width of fire spreading kernel
$\sigma _G$	5	m	width of spontaneous fire kernel

**Fig. 1:**
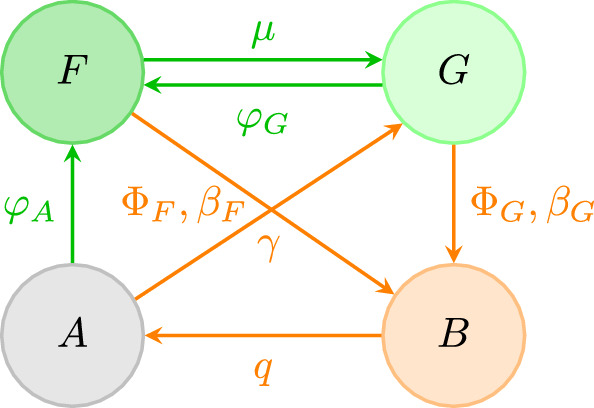
State transition diagram of the spatial FGBA model. Transition arrows are labeled with the relevant parameters and/or flammability functions. Forest and grass/fire timescale transitions are shown in green and orange, respectively

There are three levels of time separation in the rate parameters given by $\beta _G,\beta _F, q > \gamma \gg \varphi _A, \varphi _G, \mu , f_0, g_0$. This concludes our mathematical description of the spatial FGBA model. We summarize the model in the state transition diagram in Fig. (1), showing the relevant parameters and functions governing transitions between states at each vegetation site.

### Mean-Field Approximations of the Spatial FGBA Model

Theorem 2.2.1 in Patterson et al. (2020) shows that the spatial-stochastic FGBA model with uniformly randomly distributed sites on $\Omega$ converges to a spatial McKean-Vlasov process in the limit as *N* (the number of sites) tends to infinity. We may think of this limiting processes as a type of spatial mean-field limit since it retains information about spatial structure, but the vegetation at each location only interacts with other locations through weighted spatial averages of all other locations. Let *X*(*r*, *t*) indicate the probability that the limiting McKean-Vlasov process at location *r* and time *t* is in state *X*. Then the forward equation governing the dynamics of the probability densities of the limiting process are given by the following system of integro-differential equations (IDEs):

2 $$\begin{aligned} \frac{d}{dt} F(r,t)&= (\varphi _G G(r,t) + \varphi _A A(r,t))\int _{\Omega }W_F(r,r')F(r',t)dr' - \mu F(r,t)\nonumber \\&\quad -\beta _F F(r,t) \int _{\Omega }W_B(r,r')B(r',t)dr' -\Phi _F\bigg [\int _{\Omega }W_G(r,r')G(r',t)dr'\bigg ] F(r,t),\nonumber \\ \frac{d}{dt}G(r,t)&= \gamma A(r,t) - \varphi _G G(r,t) \int _{\Omega }W_F(r,r')F(r',t)dr' +\mu F(r,t) \nonumber \\&\quad -\Phi _G\bigg [\int _{\Omega }W_G(r,r')G(r',t)dr'\bigg ] G(r,t) - \beta _G G(r,t) \int _{\Omega }W_B(r,r')B(r',t)dr',\nonumber \\ \frac{d}{dt}B(r,t)&= ( \Phi _F F(r,t) + \Phi _G G(r,t) )\bigg [\int _{\Omega }W_G(r,r')G(r',t)dr'\bigg ] - qB(r,t) \nonumber \\&\quad + (\beta _G G(r,t) + \beta _F F(r,t)) \int _{\Omega }W_B(r,r')B(r',t)dr',\nonumber \\ \frac{d}{dt} A(r,t)&= q B(r,t) - \gamma A(r,t) - \varphi _A A(r,t) \int _{\Omega }W_F(r,r')F(r',t) dr', \end{aligned}$$

for each $r \in \Omega$. Moreover, $F(r,t)+G(r,t)+B(r,t)+A(r,t) = 1$ for each $(r,t)\in \Omega \times \mathbb {R}_+$.

To gain a rough, intuitive understanding of the stochastic FGBA model’s dynamics, we will analyze a more analytically tractable nonspatial mean-field model derived from (2) that takes the form of a system of ordinary differential equations. The general structure of the solution space of the spatial model (2) is not analytically tractable. One can investigate the linear stability of spatially homogeneous steady-state solutions, but we expect similar stability criteria to the nonspatial mean-field case for most reasonable choices of the kernel functions, as has been shown for related forest-savanna models posed in this framework (Patterson et al. 2024). Using the nonspatial mean-field approximation results as a baseline will also allow us to isolate the impact of spatial structure in the stochastic FGBA model. In addition to spatial structure, the stochastic FGBA model allows us to retain stochastic, discrete and finite-size effects, which we expect to be especially important for short-time and transient dynamics (Hastings et al. 2018), which we can contrast against the nonspatial mean-field model.

To obtain a nonspatial (spatially implicit) mean-field model from (2), we assume that the types of states are well-mixed throughout the spatial domain $\Omega$ so that interactions depend only on the fractions of land occupied by each state. One way to achieve this is to take the kernels functions to be uniform on $\Omega$ and then define the land cover proportions $F(t):= \int _\Omega F(r',t)dr'/\text {area}(\Omega )$, $G(t):= \int _\Omega G(r',t)dr'/\text {area}(\Omega )$, and so on. The system (2) then simplifies to the system of ordinary differential equations:

3 $$\begin{aligned} {\left\{ \begin{array}{ll} \frac{d}{dt}F(t) = (\varphi _G G + \varphi _A A) F -\beta _F B F -\Phi _F(G)F- \mu F \\ \frac{d}{dt} G(t) = \gamma A +\mu F- \varphi _GFG -\Phi _G(G) G - \beta _GBG \\ \frac{d}{dt} B(t) =\Phi _G(G) G + \Phi _F(G)F + (\beta _G G + \beta _F F)B -q B\\ \frac{d}{dt} A(t) = q B - \gamma A - \varphi _AF A. \end{array}\right. } \end{aligned}$$

**Fig. 2:**
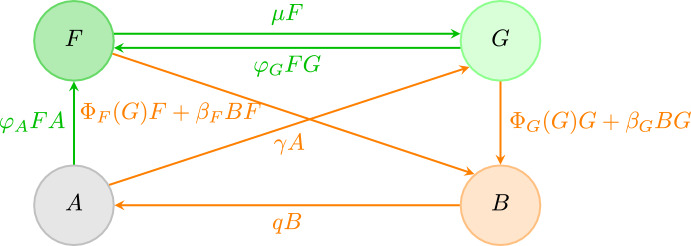
FGBA model state transition diagram in the mean-field limit. Transition arrows are labeled by the transition rates. Forest and fire timescale transitions are shown in green and orange, respectively

The state transition diagram for the spatial FGBA model in the mean field limit is illustrated in Fig. (2). First notice that due to the $G \xrightarrow {\Phi (G)G} B \xrightarrow {qB} A \xrightarrow {\gamma A} G$ cycle then if any one of $\overline{G}, \overline{B}$, or $\overline{A}$ is nonzero then all three must be nonzero. Next, since there is an $F \xrightarrow {\mu F}G$ transition then $\overline{F} > 0$ implies $\overline{G} > 0$. Thus, we can classify all steady states as either GBA (where $\overline{F} = 0$ and $\overline{G}, \overline{B}, \overline{A} \ne 0$) or FGBA (where $\overline{F}, \overline{G}, \overline{B}, \overline{A} \ne 0$).

## Analysis of the nonspatial FGBA model

Before studying the full spatial stochastic FGBA model introduced above, we perform some qualitative analysis of solutions to the nonspatial mean-field system (3).

### GBA Steady States

As it is the simplest subsystem of the model, we first consider the case when forest is absent from the landscape. Set $F = 0$ and use the relation $A = 1 - G - B$ to eliminate *A* from (3) to obtain

4 $$\begin{aligned} {\left\{ \begin{array}{ll} \frac{d}{dt}G(t) & = \gamma (1-G-B) - \Phi _G(G) G- \beta _GBG \\ \frac{d}{dt} B(t) & = \Phi _G(G)G + \beta _G GB - qB. \end{array}\right. } \end{aligned}$$

Establishing the forward invariance of the GBA subspace is straightforward, and so the proof of the following proposition is deferred to Appendix A.

#### Proposition 1

For any ecologically relevant initial value (i.e. $G(0) \ge 0$, $B(0) \ge 0$, $G(0) + B(0) \le 1$), the solution (*G*(*t*), *B*(*t*)) for equations (4) remains ecologically relevant for all times $t \in \mathbb {R}_+$.

Next, we solve for the steady-state solutions of the model by setting both equations in the system (4) equal to 0. Rearranging the first equation in (4), shows that

$$ \overline{B} = \frac{\gamma (1-\overline{G})}{\gamma + \beta _G \overline{G}} - \frac{\Phi _G(\overline{G})\overline{G}}{\gamma + \beta _G \overline{G}}. $$

Insert this relation into the second equation in (4) and multiply across by $\gamma + \beta _G \overline{G}$ to obtain

$$ 0 = \Phi _G(\overline{G})\overline{G} \left( \gamma + \beta _G \overline{G} \right) +\beta _G \gamma (1-\overline{G})\overline{G} - \beta _G \Phi _G(\overline{G})\overline{G}^2 -q \gamma (1-\overline{G}) + q \Phi _G(\overline{G})\overline{G}. $$

Rearranging the equation above shows that the steady-state solution $\overline{G}>0$ obeys:

5 $$\begin{aligned} \Phi _G(\overline{G})&= (1-\overline{G})\frac{\gamma q}{\gamma + q} \bigg (\frac{1}{\overline{G}}-\frac{\beta _G}{q}\bigg ). \end{aligned}$$

Unfortunately, closed-form expressions for the equilibria cannot be obtained except in special cases due to the sigmoidal properties of the function $\Phi _G$. However, we can readily deduce the uniqueness of the steady state. This analysis naturally involves studying properties of the function

6 $$\begin{aligned} \mathcal {F}(G) := (1-G)\frac{\gamma q}{\gamma + q} \bigg (\frac{1}{G}-\frac{\beta _G}{q}\bigg ), \quad G \in (0,1), \end{aligned}$$

which is the right-hand side of equation (5).

#### Proposition 2

For any positive parameter values $\gamma , q,$ and $\beta _G$ there exists a unique GBA steady state.

#### Proof of Proposition 2

By definition, $\overline{G}$ is a steady state if and only if $\mathcal {F}(\overline{G}) = \Phi _G(\overline{G})$. Let us first consider the case where $q < \beta _G$. Then in the interval [0, 1], $\mathcal {F}$ has exactly two roots located at $\frac{q}{\beta _G}$ and 1. Furthermore, $\mathcal {F} > 0$ on $(0, q/\beta _G)$ and $\mathcal {F} < 0$ on $(q/\beta _G,1)$. Since $\Phi _G > 0$ on (0, 1] and $0 \le \Phi _G(0) \le \infty$ then any roots of $\mathcal {F} - \Phi _G$ in [0, 1] must occur in the interval $(0, q/\beta _G)$. Next note that the derivative of $\mathcal {F}$ is

$$\begin{aligned} \mathcal {F}'(G) = \frac{\gamma q}{\gamma + q}\bigg (\frac{\beta _G}{q} - \frac{1}{G^2} \bigg ) \end{aligned}$$

and has a single root in [0, 1] located at $\sqrt{q/\beta _G}$. Furthermore, $\mathcal {F}' < 0$ on $(0, \sqrt{q/\beta _G})$. Since $(0, q/\beta _G) \subset (0, \sqrt{q/\beta _G})$ it follows that $\mathcal {F}$ is strictly monotonically decreasing on $(0, q/\beta _G)$. Next since $\Phi _G$ is monotonically increasing on $(0, q/\beta _G)$ then $\mathcal {F} - \Phi _G$ is strictly monotonically decreasing on $(0, q/\beta _G)$. Since $\mathcal {F} - \Phi _G$ is continuous on $\mathbb {R}^+$ and $\lim _{G \rightarrow \infty }(\mathcal {F} - \Phi _G)(G) = +\infty$ while $(\mathcal {F}- \Phi _G)(q/\beta _G) = -\Phi _G(q/\beta _G) < 0$ then $\mathcal {F} - \Phi _G$ must have exactly one root in $(0, q/\beta _G)$. It follows that there is a unique steady state $\overline{G} \in [0,1]$.

**Fig. 3:**
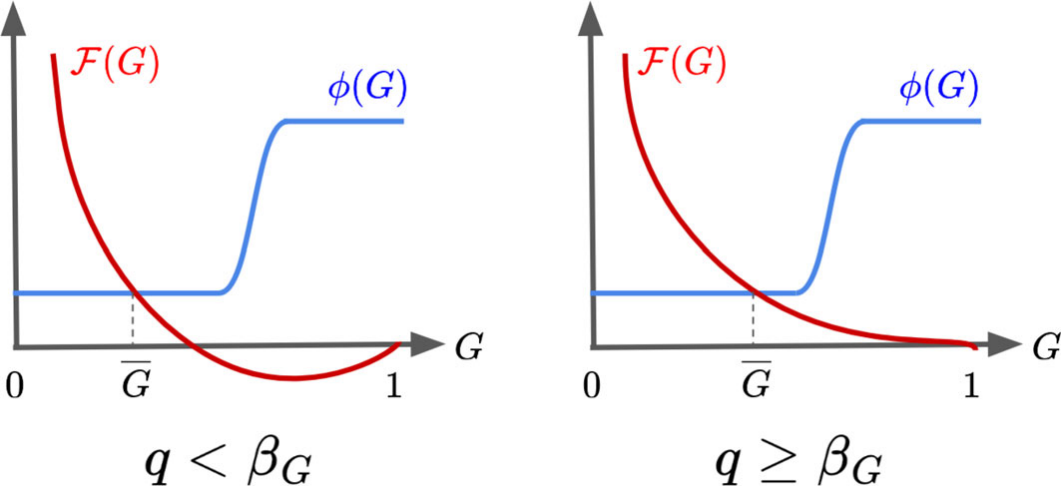
Plots of $\mathcal {F}$ and $\Phi _G$ (denoted by $\phi$) in the $q < \beta _G$ case (left) and the $q \ge \beta _G$ case (right)

When $q \ge \beta _G$, $\mathcal {F}$ has exactly one root at 1 in the interval [0, 1] and $\mathcal {F} > 0$ on (0, 1). By the same argument as before, $\mathcal {F}' < 0$ on $(0,\sqrt{q/\beta _G})$, so $\mathcal {F}$ is strictly monotonically decreasing on (0, 1). The rest of the argument is the same as the $q < \beta _G$ case with $q/\beta _G$ replaced by 1. Thus, a unique GBA steady state always exists for any choice of system parameters. See Fig. (3) below for a graphical illustration.

$$\square $$

Graphically, from Fig. (3), we note that $\overline{G}$ increases as $\frac{\gamma q}{\gamma + q}$ increases. Noting that $\partial _{\gamma }(\frac{\gamma q}{\gamma + q}) > 0$ and $\partial _{q}(\frac{\gamma q}{\gamma + q}) > 0$ when $\gamma , q > 0$ it follows that increasing either $\gamma$ or *q* will increase the proportion of grass in the steady state. This is logical since increasing $\gamma$ allows faster regrowth of grass from ash, and increasing *q* allows faster fire quenching into ash from which grass can regrow. Similarly, increasing $\frac{q}{\beta _G}$ also increases $\overline{G}$. This also follows intuition: the case of increasing *q* has already been discussed, while decreasing $\beta _G$ reduces the rate at which grass burns due to fire spread. We also note that increasing $g_0$ or $g_1$ reduces $\overline{G}$ as expected, since doing so increases the flammability of grass. Lastly, we note that the presence of the GBA steady state depends on the fact that $\Phi _G$ is non-decreasing. Thus, if $\Phi _G$ were constant, the result of the system containing a single unique GBA steady state would still hold.

#### Stability of the GBA Steady State

In this section, we demonstrate two results regarding the stability of the GBA steady state. The first concerns stability within the GBA subsystem, and the second gives an interpretable condition characterizing when we can expect the GBA subsystem to be stable to forest invasion.

##### Theorem 3

The GBA steady state is stable to perturbations within the $F=0$ subspace for any parameter values.

##### Proof

We compute the linearization matrix at $F = 0$ of the system (4), which yields

$$\begin{aligned} {\textbf {J}}(F,\,G,\,B)\vert _{F=0} = \begin{bmatrix} -\gamma -\Phi _G'(G)G-\Phi _G(G)-\beta _G B,~\,\,\,\, & -\gamma - \beta _G G \\ \Phi _G'(G) G + \Phi _G(G) + \beta _G B, & -q + \beta _G G \end{bmatrix}. \end{aligned}$$

The fixed point $(\overline{G}, \overline{B})$ is stable to perturbations within the $F = 0$ boundary exactly when $J_{F=0}(\overline{G}, \overline{B})$ has eigenvalues with strictly negative real parts. By the Routh-Hurwitz stability criteria, this occurs if and only if ${{\,\textrm{Tr}\,}}({\textbf {J}}_{F=0}(\overline{G}, \overline{B})) < 0$ and $\det ({\textbf {J}}_{F=0}(\overline{G}, \overline{B})) >0$ (Edelstein-Keshet 2005). Note that since $\overline{G} < \frac{q}{\beta _G}$ then $-q + \beta _G \overline{G} < 0$ so ${{\,\textrm{Tr}\,}}(J_{F=0}(\overline{G}, \overline{B})) < 0$ is clearly satisfied. The condition $\det ({\textbf {J}}_{F=0}(\overline{G}, \overline{B})) >0$ is equivalent to

$$\begin{aligned} \Phi '(\overline{G}) > \frac{\gamma q}{(\gamma + q)^2}\bigg (\frac{\beta }{q}-\frac{1}{\overline{G}^2} \bigg ) = \mathcal {F}'(\overline{G}), \end{aligned}$$

which holds from graphical inspection or more rigorously by recalling from our earlier argument that $\Phi _G'(\overline{G}) \ge 0$ while $\mathcal {F}'(\overline{G}) < 0$. $\square$

##### Proposition 4

The GBA steady state is stable to invasions by *F* exactly when

7 $$\begin{aligned} \beta _F \overline{B} + \Phi _F(\overline{G}) + \mu > \varphi _G \overline{G} + \varphi _A \overline{A}. \end{aligned}$$

##### Proof

We compute the linearization matrix for the full FGBA system and evaluate it at $F = 0$. We use $A = 1 - F - G - B$ to obtain a dynamical system in the three variables *F*, *G*, and *B* which gives the matrix

$$\begin{aligned}&{\textbf {J}}(F,\, G,\, B)\vert _{F = 0} \\&= \begin{bmatrix} \varphi _G G + \varphi _A A - \beta _F B -\Phi _F(G) - \mu , & 0, & 0 \\ -\gamma -\varphi _G G +\mu , & -\gamma -\Phi _G'(G)G-\Phi _G(G)-\beta _G B,~ & -\gamma - \beta _G G \\ \Phi _F(G) + \beta _F B, & \Phi _G'(G) G + \Phi _G(G) + \beta _G B, & -q + \beta _G G \end{bmatrix}. \end{aligned}$$

The GBA steady state is stable to invasion by *F* if all the eigenvalues of ${\textbf {J}}(\overline{F}, \overline{G},\overline{B})\vert _{\overline{F} = 0}$ have negative real parts. We have already shown that the eigenvalues of the $2 \times 2$ submatrix in the lower right have strictly negative real parts so the GBA steady state is stable if and only if the entry in the upper left is negative. $\square$

An intuitive interpretation of equation (7) is that the GBA equilibria is resistant to invasion by forest when the rate of tree mortality by fire spread through forest, spontaneous forest fires, and natural mortality (left-hand side) exceeds the rate of forest seeding into the steady state grass and ash land (right-hand side). Since a unique GBA steady state exists for any value of the system parameters, but its stability can vary based on the parameter values, we expect to find transcritical bifurcations within the parameter space when equality occurs in equation  (7). In other words, a stable GBA steady state bifurcates into an unstable no-forest state and a stable forest state.

Some example bifurcation diagrams in $\beta _G$, $\beta _F$, $\varphi = \varphi _G = \varphi _A$ and $\mu$ using partially timescale-separated parameter values are given in Fig. (6). As expected, when $\varphi$ is increased past a critical value or when $\beta _G$, $\beta _F$, and $\mu$ are decreased past a critical value, the proportion of forest sites within $\Omega$ becomes non-zero. However, the parameter values produce a mostly ash grassland steady state which is not very ecologically realistic. Nonetheless, it will later be shown that the spatial FGBA model has grassy steady states distinct from the ash-dominated, spatially homogeneous GBA steady state.

#### Estimates of the GBA Steady State

We will now show that using our estimates of the parameter values, the steady state $(\overline{G}, \overline{B}, \overline{A})$ can be easily estimated. Notice that $\mathcal {F}(G)$ becomes asymptotic to $\frac{\gamma q}{\gamma + q} (\frac{1}{G} - \frac{\beta _G}{q})$ as $G \rightarrow 0$ and the coefficient $\frac{\gamma q}{\gamma + q}$ is of order 2. Since $\Phi _G(G)$ is of order 0 or lower for $G \in [0,1]$ then we can approximate the condition $\Phi _G(G) = \mathcal {F}(G)$ as $0 = \mathcal {F}(G)$ which gives $\overline{G} \approx \frac{q}{\beta _G}$. It follows that to increase the portion of land covered in grass, we must either increase *q*, i.e. increasing the fire quenching rate, or decrease $\beta _G$, i.e. decreasing the rate of fire spread through grass, both of which make sense intuitively. By solving System 4 and using $G+B+A=1$ we can compute an estimate of the GBA steady state as well as the ratio $\overline{B}/\overline{A}$:

8 $$\begin{aligned} (\overline{G}, \overline{B}, \overline{A}) \approx \bigg (\frac{q}{\beta _G}, \frac{\gamma (1-\frac{q}{\beta _G})}{q+\gamma }, \frac{q(1 - \frac{q}{\beta _G})}{q+\gamma }\bigg ) \Longleftrightarrow \frac{\overline{B}}{\overline{A}} \approx \frac{\gamma }{q}. \end{aligned}$$

Based on the estimate, the ratio between the portions of land in burning and ash states is determined by $\gamma$, the rate of grass regrowth from ash, and *q*, the rate of fire quenching in a manner that agrees with intuition.

Then Eq. (8) can be used to give Eq. (7) purely in terms of the model parameters:

$$\begin{aligned} \frac{\beta _F \gamma (1 - \frac{q}{\beta _G})}{q+\gamma } + \Phi _F\Big (\frac{q}{\beta _G}\Big ) + \mu > \frac{q\varphi _G}{\beta _G} + \frac{q\varphi _A(1-\frac{q}{\beta _G})}{q+\gamma }. \end{aligned}$$

In particular, if we vary each of the parameters $\beta _F, \gamma , f_0, \mu ,$ and $\varphi = \varphi _A = \varphi _G$ one at a time and fix all other parameters at the estimates given in Table 2, we can compute the transcritical bifurcation points for each parameter. To simplify the calculations, we will treat $\Phi _F$ as a Heaviside step function, that is

$$\begin{aligned} \Phi _F(x) = f_0 + (f_1-f_0)H(x - \theta ), \quad \text { where } \quad H(x) = {\left\{ \begin{array}{ll} 1 & x \ge 0, \\ 0 & x < 0, \end{array}\right. } \end{aligned}$$

which can be viewed as $\Phi _F$ in the limit $s_F \rightarrow 0$.

### FGBA Model Steady States

We next solve for the FGBA steady states. We first use the relation $A = 1 - F -G - B$ to eliminate the ash land cover fraction from (3), which gives the following system of equations:

9 $$\begin{aligned} \frac{d}{dt}F(t)&= (1-F-B) \varphi F -\beta _F B F -\Phi _F(G)F- \mu F \end{aligned}$$

10 $$\begin{aligned} \frac{d}{dt}G(t)&= \gamma (1-F-G-B) +\mu F- \varphi FG -\Phi _G(G) G - \beta _GBG \end{aligned}$$

11 $$\begin{aligned} \frac{d}{dt} B(t)&=\Phi _G(G) G + \Phi _F(G)F + (\beta _G G + \beta _F F)B -q B . \end{aligned}$$

From this point forward, we will set $\varphi = \varphi _A = \varphi _G$ to simplify calculations. This is an ecologically reasonable assumption since the rate of forest spread into ash should not differ substantially from the rate of forest spread into grassland. Note that a similar assumption was made in Wuyts and Sieber (2023). Then after setting $\dot{G}= \dot{B}= \dot{A} = 0$ we can solve for roots $(\overline{F}, \overline{G}, \overline{B}, \overline{A})$. As for the GBA subspace, the full model retains forward invariance of the ecologically relevant region of the phase-space (proof deferred to Appendix A).

#### Proposition 5

For any ecologically relevant initial value (i.e. $F(0) \ge 0$, $G(0) \ge 0$, $B(0) \ge 0$, $F(0) + G(0) + B(0) \le 1$), the solution (*F*(*t*), *G*(*t*), *B*(*t*)) remains ecologically relevant for all times $t \in \mathbb {R}$.

We can now investigate the transcritical bifurcation anticipated to occur at the transition from the GBA steady state to states with non-zero forest. Let $\overline{B}(F)$ be a function that outputs the equilibrium burning proportion(s) *B* for any given input *F*. The function $\overline{B}(F)$ can be obtained from Eqs. (9) to (11) by solving for *B* in terms of *F* after setting $\dot{G}= \dot{B}= 0$ and enforcing $F + G+ B+ A = 1$. In general, $\overline{B}(\cdot )$ is a complicated function we will analyze later. The vector field for forest cover can be expressed as

$$\begin{aligned} Q(F)&= (1 - F - \overline{B}(F))F \varphi - \beta _F \overline{B}(F)F - (f_1 + \mu )F \\&= -\overline{B}(F) F (\varphi +\beta _F) - \varphi F^2 + F(\varphi -\tilde{\mu }) \end{aligned}$$

where we introduce the parameter $\tilde{\mu }:= \mu + f_1$ to reduce notational clutter. The normal form for a transcritical bifurcation is $\dot{x}=a_1x+x^2$. To verify that the genericity conditions for a transcritical bifurcation hold, we first note that $Q(F = 0) = 0$ as expected since $F=0$ is an equilibrium. Furthermore,

$$\begin{aligned} Q'(F = 0)&= -\overline{B}(0)(\varphi + \beta _F) + \varphi - \tilde{\mu } \end{aligned}$$

which indicates the stability of the $F = 0$ equilibrium (i.e. the *GBA* equilibrium). For instance, note that the inequality $Q'(F) < 0$ is exactly the same as Eq. (7) after making the simplifications $\varphi = \varphi _G = \varphi _A$ and $\Phi _F(\overline{G}) = f_1$; the latter assumption is motivated by assuming we are in the high-fire incidence regime with grass the dominant cover type (see Table 2 for the definition of the parameter $f_1$). Lastly, we have

$$\begin{aligned} Q''(0)&= - 2\overline{B}'(0)(\varphi +\beta _F) -2\varphi \end{aligned}$$

and it follows that as long as $Q''(0) \ne 0$ a non-degenerate transcritical bifurcation occurs at parameter values where $Q'(F=0)$ i.e. when

12 $$\begin{aligned} \overline{B}(0)&= \frac{\varphi - \tilde{\mu }}{\varphi + \beta _F} \end{aligned}$$

Next, we know that after passing the transcritical bifurcation, the system displays two distinct equilibria, one stable and the other unstable. Since $F=0$ is always an equilibrium, it remains to be determined whether or not the second equilibrium is ecologically plausible, i.e., contained in $\mathcal {F}_{FGBA}$. To do this, we first note that the nonzero forest equilibrium can be calculated from Eq. (9) by dividing out the factor of *F* and setting $\dot{F} = 0$. This gives an implicit equation

$$\begin{aligned} \overline{F}(\overline{B})&= \frac{(\varphi -\tilde{\mu }) - (\varphi + \beta _F)\overline{B}(\overline{F})}{\varphi }. \end{aligned}$$

Next we can expand about the equilibria by writing $F = 0 \rightarrow \delta F$ while $\varphi \rightarrow \varphi + \delta \varphi$ or $\beta _F \rightarrow \beta _F + \delta \beta _F$ or $\tilde{\mu } \rightarrow \tilde{\mu }+ \delta \tilde{\mu }$. Then, after keeping only first-order terms, we find that

$$\begin{aligned} \bigg [\frac{\varphi }{\varphi + \beta _F} + \overline{B}'(0)\bigg ] \delta F = \bigg [ \frac{\tilde{\mu } + \beta _F}{(\varphi + \beta _F)^2}\bigg ] \delta \varphi = \bigg [ \frac{\tilde{\mu } - \varphi }{(\varphi + \beta _F)^2}\bigg ] \delta \beta _F = \bigg [\frac{-1}{(\varphi + \beta _F)^2}\bigg ] \delta \tilde{\mu }. \end{aligned}$$

Then, the new equilibria emerging at the transcritical point after perturbation of a parameter value is ecologically plausible exactly when $\delta F > 0$. Thus, whether or not the non-zero forest equilibria are ecologically plausible can be determined based on the parameter values $\tilde{\mu }, \beta _F, \varphi$ as well as $\overline{B}'(0)$.

Next, after some calculations (see Appendix B for details), we find that the parameter values along which the transcritical bifurcation occurs can be determined to various corrections in orders of $g_1$:

13 $$\begin{aligned} \frac{\varphi - \tilde{\mu }}{\varphi + \beta _F} = \frac{\gamma (\beta _G - q)}{\beta _G(q+\gamma )} + \frac{q g_1}{\beta _G(\beta _G - q)} + \frac{q(q+\gamma )g_1^2}{(q-\beta _G)^3 \gamma } + \mathcal {O}(g_1^3) \end{aligned}$$

**Fig. 4:**
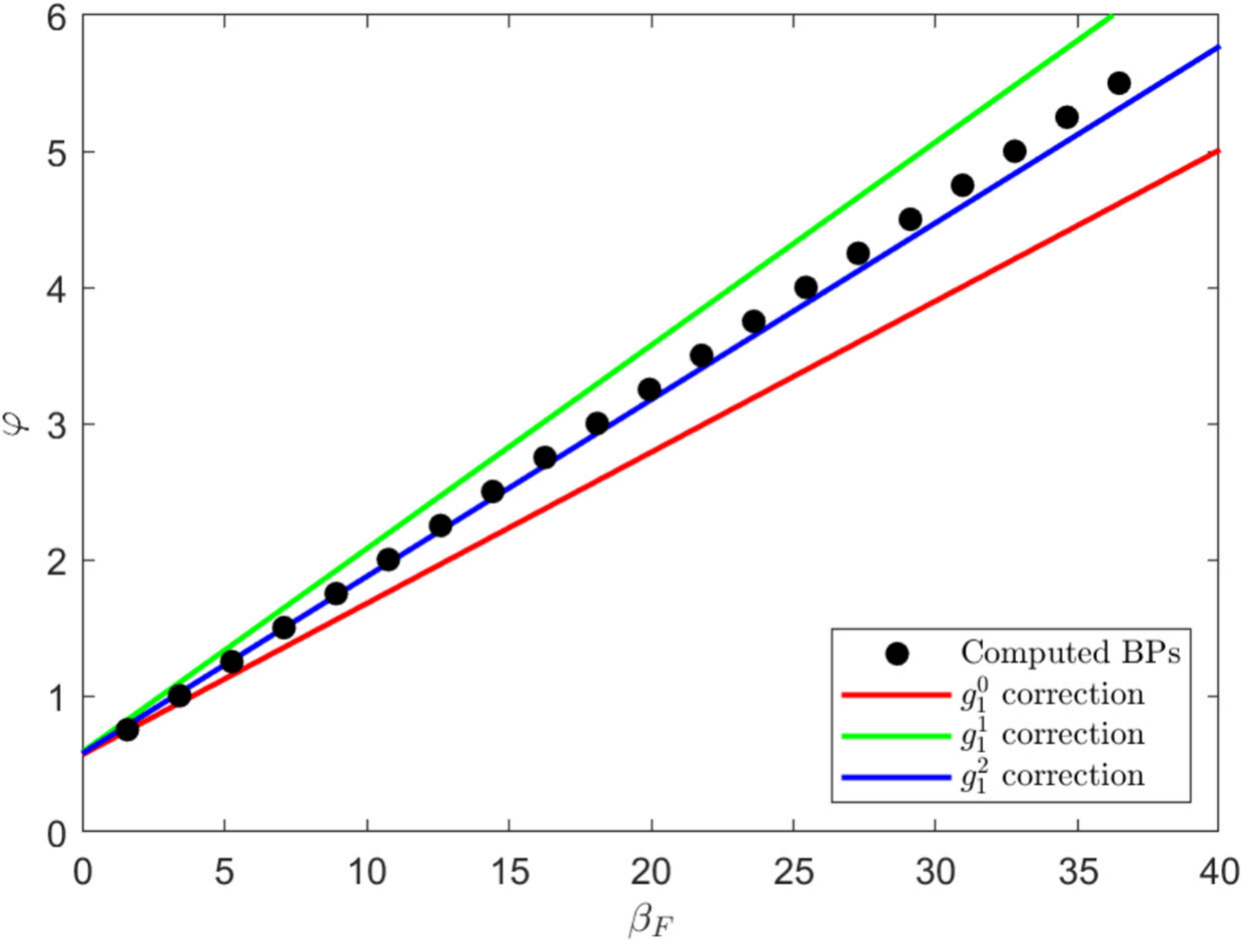
A plot of the computed branch points governing the stable to unstable transition for the GBA steady state for system (4) alongside the analytically predicted branch points in Eq. (13). Branch points are plotted as a function of $\beta _F$, the rate of fire spread over forest, and $\varphi$, the rate of forest spreading over grass and ash (assuming $\varphi = \varphi _A = \varphi _G$). Parameter values: $\beta _G = 50$, $\beta _F = 10$, $q = 30$, $\gamma = 10$, $\varphi = 0.1$, $f_0 = g_0 = 0.01$, $f_1 = 0.5$, $g_1 = 1$

A plot of numerically computed branch points and Eq. (13) plotted to various orders in $g_0$ and $f_0$ is given in Fig. (4).

#### Bifurcation diagrams (partially timescale separated)

As the previous section shows, analyzing the FGBA system with a non-zero forest is complex. As such, a numerical study of the system is helpful for a more general understanding of the system’s behavior. Numerical continuation computations with timescale-separated parameter values are extremely slow and often challenging to perform. Thus, we first carried out our analysis with a set of “partially timescale-separated parameter values”. By partially timescale-separated, we mean that the values used likely underestimate the speed at which fire dynamics operate relative to vegetation dynamics and related processes (e.g., seed dispersal). This analysis can still provide valuable insight into overall system dynamics and serve as a baseline for further analysis of scenarios with more realistic levels of timescale separation (see Section 4).

**Fig. 5:**
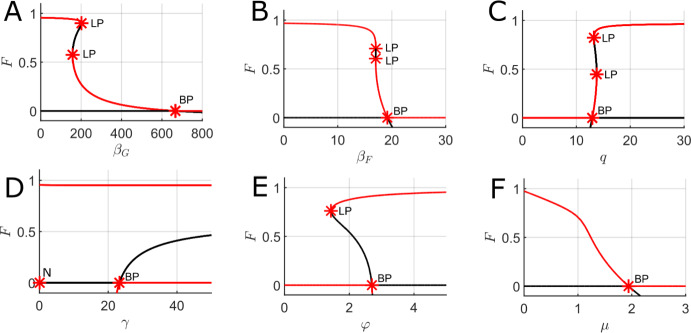
One-parameter bifurcation diagrams for the mean-field FGBA system (3) with partially timescale-separated parameter values with the forest proportion of the solution of the y-axis. A. Varying the fire spread rate over grass ($\beta _G$). B. Varying the fire spread rate over forest ($\beta _F$). C. Varying the rate at which fire is quenched. D. Varying the rate at which grass regrows from ash ($\gamma$). E. Varying the rate of forest spread over ash and grass ($\varphi$). F. Varying the rate of tree mortality due to non-fire-related causes ($\mu$). Red lines indicate stable equilibria, while black lines indicate unstable equilibria. LP indicates a saddle-node bifurcation point (limit point), BP indicates a transcritical bifurcation point (branch point), and N indicates a neutral saddle equilibrium with no stability change. Parameter values (fixed if not the bifurcation parameter): $\beta _G = 50$, $\beta _F = 10$, $q = 30$, $\gamma = 10$, $\varphi = \varphi _A = \varphi _G = 1$, $\mu = 0.01$, $f_0 = g_0 = 0.01$, $f_1 = 0.5$, and $g_1 = 1$

Fig. (5) below illustrates the presence or absence of forest from stable steady-states of the FGBA model through one-parameter bifurcation diagrams. In each panel, the forest proportion is plotted on the y-axis against a different system parameter on the x-axis, with solid red lines indicating stable equilibria and solid black lines denoting unstable equilibria. Fig. (5)ABCDE all contain parameter regimes with multiple stable equilibria (bistability), with the only parameter not showing a bistable range being Fig. (5)F, which varies the tree mortality rate due to non-fire-related causes ($\mu$). These bistable ranges are often marked by saddle node bifurcations (denoted LP in the diagrams) at which new equilibria emerge or vanish, such as in Fig. (5)ABC. As expected, the zero forest cover state is stable at low values of the rate of forest spread into grass and ash ($\varphi = \varphi _A = \varphi _G$) and high values of the rate of fire spread into forest ($\beta _F$). Another generic feature of these one-parameter bifurcation diagrams is the change of stability of the GBA subsystem via transcritical bifurcations (denoted BP in the diagrams) at which forest either enters or is excluded from the system. For example, in Fig. (5)D there is a stable forest-dominant steady-state for all values of the rate at which grass regrows from ash ($\gamma$), and bistability emerges when $\gamma$ reaches a critical value ($\approx 22$) at which the GBA steady state becomes stable. Similarly, a transcritical bifurcation is present at the critical parameter values of both $\varphi$ and $\beta _F$ where the GBA steady state changes stability (Fig. (5)BE). Additional two-parameter bifurcation analysis illustrating the size of the bistable regions as multiple parameters are varied together can be found in Appendix C.

**Fig. 6:**
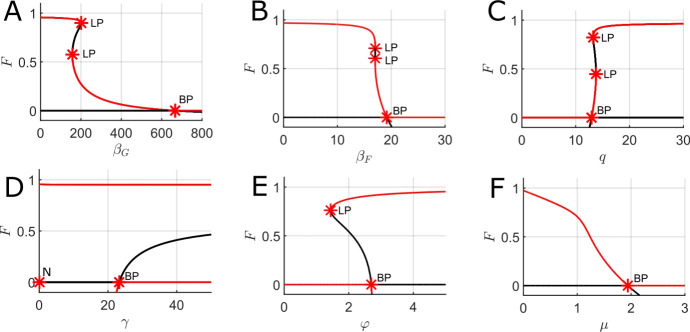
Equilibrium land state proportions (i.e., steady-state values) of the mean-field FGBA system (3) reached starting from a low forest state ($F=0.01$ initial condition) for partially timescale separated parameter values varying one system parameter at a time. A. Varying the fire spread rate over grass ($\beta _G$). B. Varying the fire spread rate over forest ($\beta _F$). C. Varying the rate of forest spread over ash and grass ($\varphi$). D. Varying the rate of tree mortality due to non-fire-related causes ($\mu$). Parameter values (fixed if not the bifurcation parameter): $\beta _G = 50$, $\beta _F = 10$, $q = 30$, $\gamma = 10$, $\varphi = 1$, $\mu = 0.01$, $f_0 = g_0 = 0.01$, $f_1 = 0.5$, and $g_1 = 1$

Fig. (6) gives more detail on the structure of the steady-states shown in Fig. (5) by once more varying model parameters systematically but now plotting all four cover type proportions on the y-axis. In each case, the system is started from a very low forest cover proportion ($F=0.01$) and run until it reaches a steady state. This serves to highlight the balance between grass, ash and burning states, and also the abrupt nature of the transitions possible when parameters are varied. For example, in Fig. (6)C, a small change in the rate of forest spread over ash and grass (around $\varphi \approx 2.8$) takes us from stable forest exclusion to a steady state in which forest occupies nearly the entire landscape.

#### Bifurcation diagrams (timescale separated)

Several one-parameter bifurcation diagrams were plotted using fully timescale-separated parameter values using MatCont (Dhooge et al. 2008) to illustrate the role of timescale separation and contrast with the results of the previous section. Due to prohibitively slow computation time, two-parameter bifurcation diagrams were not computed for fully timescale-separated parameter values.

**Fig. 7:**
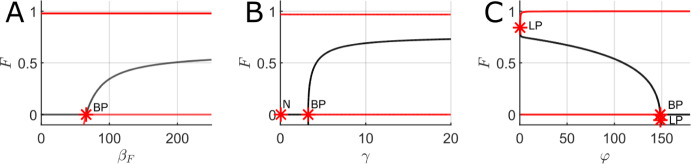
One-parameter bifurcation diagrams for the mean-field FGBA system (3) for fully timescale-separated parameter values with the forest proportion, *F*, on the y-axis and the parameter being varied on the x-axis. A. Varying the fire spreading rate in forest ($\beta _F$). B. Varying the grass regrowth rate from ash ($\gamma$). C. Varying the forest spreading rate into ash and grass ($\phi$). Red lines indicate stable equilibria while black lines indicate unstable equilibria. LP indicates a limit point, BP indicates a branch point, and N indicates a neutral saddle equilibrium. Parameter values (fixed if not the bifurcation parameter): $\beta _G = 50000$, $\beta _F = 10000$, $q = 20000$, $\gamma = 500$, $\varphi = 1$, $\mu = 0.01$, $f_0 = g_0 = 0.01$, $f_1 = 0.05$, and $g_1 = 0.1$

**Fig. 8:**
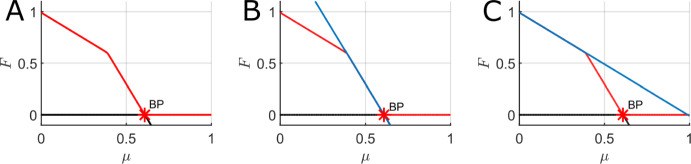
A. One parameter bifurcation diagrams for the mean-field FGBA system (3) with the forest tree mortality rate ($\mu$) as the bifurcation parameter. Red lines indicate stable equilibria while black lines indicate unstable equilibria. BP indicates a branch point. B. Same as panel A with the linear regime near $F=0$ overlaid as a solid blue line. C. Same as panel A with the linear regime near $F=1$ overlaid as a solid blue line. Parameter values (fixed if not the bifurcation parameter): $\beta _G = 50000$, $\beta _F = 25$, $q = 20000$, $\gamma = 500$, $\varphi = 1$, $\mu = 0.01$, $f_0 = g_0 = 0.01$, $f_1 = 0.05$, and $g_1 = 0.1$

Fig. (7) shows one-parameter bifurcation diagrams for the forest steady-state as different system parameters are varied in the mean-field FGBA model (3) for the fully timescale separated parameter set (full parameter set included in the figure caption). We immediately note the stark differences between Fig. (7)A and Fig. (5)B, which both vary the fire spread rate over forest ($\beta _F$), but with very different levels of timescale separation between fire and vegetation dynamics. For the more realistic parameter set used in Fig. (7)A, we observe a qualitatively different picture of the forest dynamics as $\beta _F$ varies; we no longer have saddle-node bifurcations and a narrow bistable parameter regime, but instead we observe bistability between the GBA steady state and a forest steady state for all $\beta _F$ sufficiently large. We note that such a significant qualitative change is not entirely unexpected since we have moved numerous parameters in shifting to the fully timescale separated parameter set and thus we do not expect Fig. (7)A to be a simple smooth deformation of Fig. (5)B. However, Fig. (7)B and Fig. (7)C do qualitatively match the corresponding partially timescale separated bifurcation diagrams (Fig. (5)D and Fig. (5)E), albeit with the locations of the saddle node and branch points significantly shifted in parameter space. Overall, the results of Fig. (7) illustrate that the degree of timescale separation can significantly alter the underlying bifurcation structure of the model and increased timescale separation tends to enlarge the bistable regions of parameter space (at least from the perspective of varying any one parameter in isolation).

Fig. (8)A shows the one-parameter bifurcation diagram for the forest steady-state as the forest tree (non-fire) mortality rate $\mu$ varies for the full timescale separated parameter set (cf. Fig. (5)F). However, the branch point occurs very close to zero so we set $\beta _F = 25$ to ensure that the $\mu$ branch point occurs at a reasonable positive value and allows us to observe the trends in *F*. The steady states curves for *F* notably has two highly linear regimes in this case. In this parameter regime, we can deduce asymptotic formulae for these curves and these asymptotic approximations are shown as solid blue lines in Fig. (8)BC. The associated calculations and formulae for these approximating curves can be found in Appendix E.

## Dynamics of the spatial stochastic FGBA model

### Simulation algorithms and codes

The spatial FGBA model was simulated in MATLAB using the Gillespie algorithm (Gillespie 1977). All code used in this project can be accessed at https://github.com/patterd2/vegetation_fire_models. Testing and validation of the simulation codes are outlined in Appendix D.

### Grassland without forest

Unless otherwise noted, all simulations in this section and the next section were run with $L = 1$ and $N = 2000$. Parameter values were identical to those used in the timescale-separated bifurcation diagram analysis, with the widths of all spreading kernels set to $\sigma _F=\sigma _B=\sigma _G=0.05$. The mean-field analysis predicts that the GBA model will exhibit a single ash-dominated stable fixed point. On longer timescales, we typically observe fluctuations about the mean-field dynamics in most parameter regimes, as expected, but the stochastic spatial model displays more complex dynamics on shorter timescales, and hence we focus on this case. Transient dynamics play a crucial role in ecological applications by capturing short- to medium-term ecosystem responses that differ from long-term equilibria, especially following disturbances or environmental change (Hastings 2001; Hastings et al. 2018). In the context of forest–savanna ecosystems, these dynamics may help to explain shifts in vegetation structure, species composition, and fire regimes observed in remotely sensed data on shorter timescales.

**Fig. 9:**
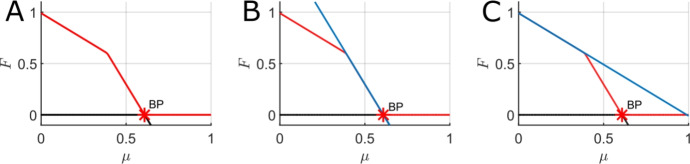
Simulations of the spatial-stochastic GBA model. A. System dynamics on the fire timescale (with time *t* in years), with the proportions of space covered by each state on the y-axis. Note that a slight increase in burning area (fire ignition) is followed by a large increase in ash and a large decrease in grass. The grass cover then gradually recovers from the ash state. B. System dynamics on a grass timescale showing repeated large fire events followed by grass recovery. C. Montage of fire ignition and spreading in grassland on a fire timescale as shown in A. D. Montage of grass regrowth following a fire ignition event in grassland on the grass timescale as shown in A. Parameter values: $\beta _G = 50000$, $\beta _F = 10000$, $q = 20000$, $\gamma = 500$, $\varphi _A=\varphi _G = 1$, $\mu = 0.01$, $f_0 = g_0 = 0.01$, $f_1 = 0.05$, $g_1 = 0.1$, $N=2000$, $L=1$, $\sigma _B=0.035$, $\sigma _F=\sigma _G=0.05$

Using timescale-separated parameter values enables the spatial-stochastic GBA model (forest excluded) to exhibit various behaviors that can only be observed appropriately on vastly differing timescales. For example, the dynamics of the cover proportions of the different states at a fire scale vs a grass time scale are shown in Fig. (9)AB. Fig. (9)A shows the profile of a single ignition event over a day, while Fig. (9)B shows multiple small fires and grass regrowth phases taking place over several months. Fig. (9)C shows a montage of the spatial progression of a typical fire ignition and spreading event occurring over a very short time (several hours).

On the fire timescale, the spatial structure of a fire front spreading through space and leaving behind a region of ash is readily apparent. On the grass timescale, the fire dynamics are obscured, but the regrowth of grass following fire spreading events is easily observed. Compared to the fire spreading, grass regrowth occurs relatively homogeneously spatially as shown in Fig. (9)D.

**Fig. 10:**
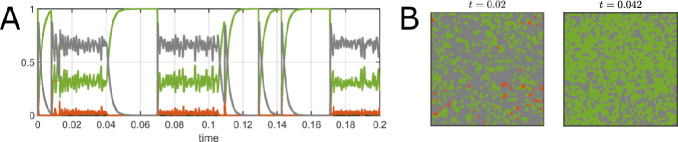
Simulations of the spatial-stochastic GBA model. A. Example of the system switching between large fires and mean-field-like behavior (i.e. fluctuations about an ash-dominated state). The y-axis is the proportion of space covered by each state and time *t* in years. B. Snapshot of the spatial system in the stochastic analogue of the ash-dominated mean-field GBA steady state at time $t=0.02$ years and a snapshot of the system in the grass-dominated regime at time $t=0.042$ years. The color legend used is shown in Fig. (9). Parameter values: $\beta _G = 50000$, $\beta _F = 10000$, $q = 15000$, $\gamma = 500$, $\varphi _A=\varphi _G = 1$, $\mu = 0.01$, $f_0 = g_0 = 0.01$, $f_1 = 0.05$, $g_1 = 0.1$, $N=2000$, $L=1$, $\sigma _F=\sigma _G=\sigma _B=0.05$

In Fig. (9)B, several ignition events occur well separated in time on the grass timescale. This suggests that, in this parameter regime, the spatial GBA model spends a lot of time in a grass-dominated regime, distinct from the (unique) mean-field steady state, consisting of mostly ash. This is an especially interesting example of long-lived transient dynamics maintaining the system away from the mean-field prediction for extended periods. The spatial model can also more closely reflect the mean-field grass steady state under certain conditions. For example, the spatial model spends significant time near the mean-field GBA steady state for certain parameter values, which allows a uniform mixing of the states across the land area. For example, setting the fire quenching rate *q* to 20000 instead of 15000 produces the behavior shown in Fig. (10)A, with the system experiencing stochastic fluctuations that cause it to switch between regimes of big fire spreading events with spatial structure and mean-field behavior. The analogue of the mean-field GBA steady state is shown in Fig. (10)B for $t=0.02$ years, while Fig. (10)B with $t=0.042$ years shows a grass-dominated regime produced by transient and spatial dynamics not present in the mean-field model. A detailed investigation of the nature of these transient phenomena is beyond the scope of the present work, but it has been noted that high-dimensionality and spatial extent can promote long transients (Hastings et al. 2018), as observed here. In particular, the grass-dominated regime in Fig. (10)B takes time to build up a sufficiently dense and continuous layer of flammable cover to facilitate the large fire required to initiate the transition back to the ash-dominated state predicted by the mean-field. We hypothesize that this mechanism plays a key role in determining the duration of these extended transients.

### Grassland with Forest

When studying grassland containing forest trees, we find additional new behavior at the forest timescale. In general, ignition events in grass cause large fires that propagate to an extent determined by the surrounding distribution of forest and destroy trees near the perimeter of the forest regions. During periods between fire events, the trees steadily regrow.

**Fig. 11:**
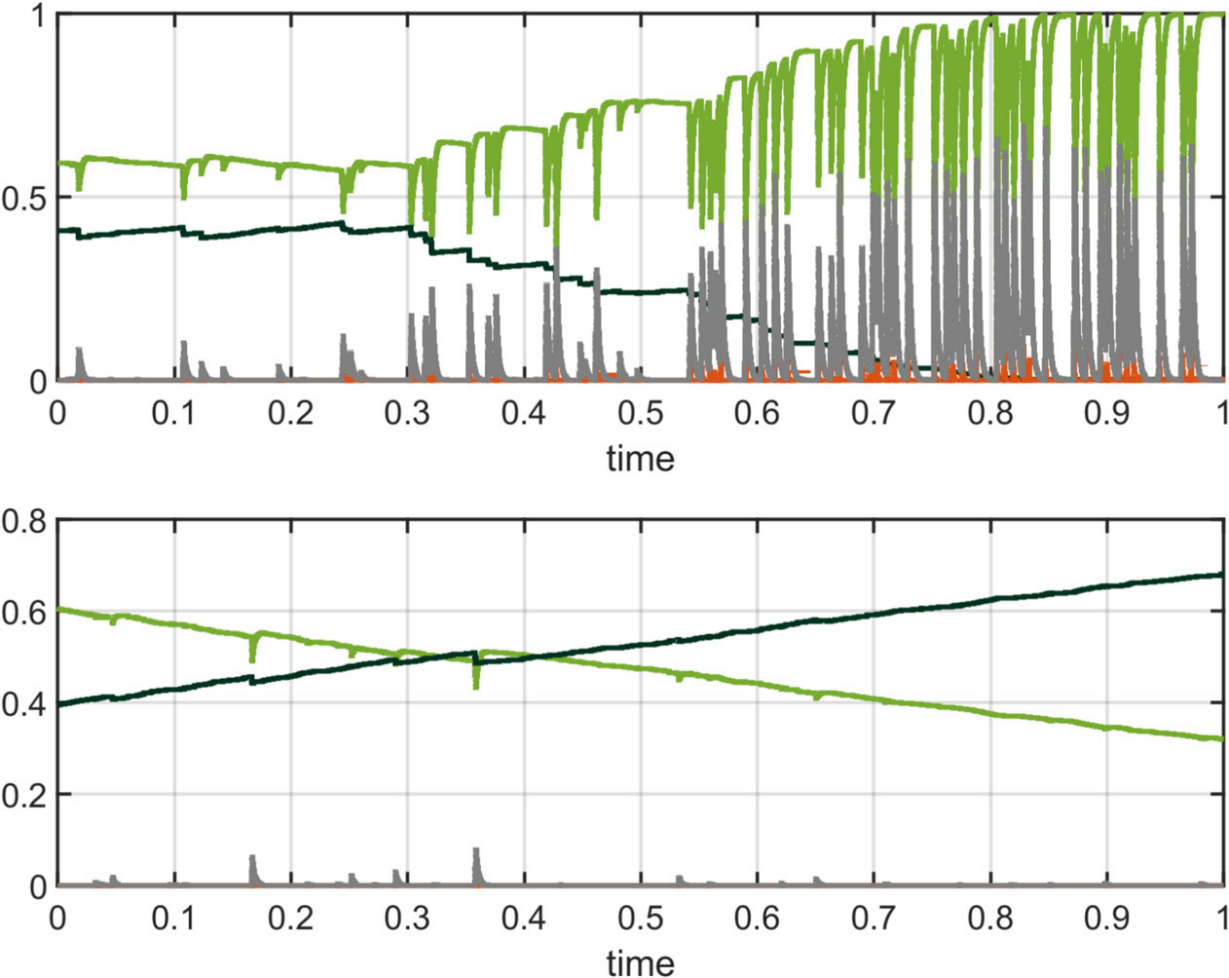
Two different possible outcomes for forest (dark green line) in the spatial-stochastic FGBA model; the proportion of space covered by each state is on the y-axis with time in years. Both simulations were run with the same parameter values and initial conditions. Forest was randomly distributed and occupied $40\%$ of sites. The remaining sites were all grass. The color legend is shown in Fig. (9). Parameter values: $\beta _G = 50000$, $\beta _F = 10000$, $q = 20000$, $\gamma = 500$, $\varphi _A=\varphi _G = 1.5$, $\mu = 0.01$, $f_0 = g_0 = 0.01$, $f_1 = 0.05$, $g_1 = 0.1$, $N=2000$, $L=1$, $\sigma _B=0.03$, $\sigma _F=\sigma _G=0.05$

In general, there are two distinct outcomes over longer time periods. In the first scenario, the forest trees grow faster than the transitions that destroy them and eventually become so dense that fires can no longer propagate; thus, the high forest state is stably maintained. Alternatively, the grass fires are sufficiently destructive to reduce the forest proportion to a vanishing or near-vanishing proportion, where the forest can no longer spread noticeably. These two cases are illustrated in Fig. (11) and confirm that our model produces the desired forest-savanna bistability in its complete spatial stochastic form. As forest cover decreases, fires become larger and more frequent.

**Fig. 12:**
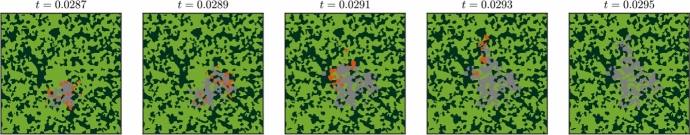
A simulation of the spatial stochastic FGBA model demonstrating the ability of forest cover to limit the extent of fire spreading and specifically to stop smaller fires from growing into larger fires on the scale of the entire spatial domain (compare to Fig. (9)CD). The color legend is shown in Fig. (9) with time *t* in years. Parameter values: $\beta _G = 50000$, $\beta _F = 10000$, $q = 20000$, $\gamma = 500$, $\varphi _A=\varphi _G = 1.5$, $\mu = 0.01$, $f_0 = g_0 = 0.01$, $f_1 = 0.05$, $g_1 = 0.1$, $N=2000$, $L=1$, $\sigma _B=0.03$, $\sigma _F=\sigma _G=0.05$

Due to the stochastic nature of the model and the underlying bistable structure, we cannot determine the long-term cover proportions at the outset; we may even observe noise-induced switching (Weinan et al. 2021) between grass and forest-dominated steady states, which cannot be understood in terms of the mean-field approximation. In simulations starting at an initial condition of high forest cover, fire size also shows a noticeable increase as the forest cover decreases. A montage demonstrating the ability of forest cover to limit the extent of fire spreading is given in Fig. (12), corresponding to the fire occurring at the start of the simulation.

The top panel of Fig. (11) highlights the dynamic nature of the open-canopy savanna-like state of the system, where frequent small to medium-scale fires are required to maintain the system state. Thus, our model in this setup could be used to distinguish between woody encroachment and natural fluctuations in the open canopy state. In contrast, we observe relatively small fluctuations in the forest-dominated state in the bottom panel of Fig. (11) and hence our initial modeling suggests that empirically observed fluctuations in forest cover are likely to be of greater concern. It may be possible to quantify this effect with more detailed parameter fitting and comparison with empirical data. However, other factors such as rainfall seasonality, which will impact fire patterns, would need to be added to the modeling structure to see how they amplify or dampen the aforementioned fluctuations (Accatino and De Michele 2013).

## Conclusions

We have developed and analyzed a spatial stochastic model appropriate for studying fire-mediated forest-savanna bistability on short timescales where stochasticity and transient dynamics are expected to play crucial roles. Our work bridges the gap between highly resolved global vegetation models and minimalist models of forest-savanna bistability, such as the original Staver-Levin model. While the extant modeling literature has often relied on mean-field approximations (Touboul et al. 2018; Staver and Levin 2012), our work highlights the need to consider appropriately detailed models when estimating ecosystem resilience and stability from empirical data. The mean-field (spatially implicit) version of our FGBA model provides a reasonably simple method to study the qualitative behavior of the system, but does not necessarily provide a full description of the dynamics of the spatial stochastic model. For instance, in the absence of forest trees, the stability of the grassland steady state in parameter regimes with sufficiently high forest mortality is observed in both the mean-field and spatial models (Figs. (5F) and (9)). However, the mean-field model predicts only a single grassland steady state at unrealistically high ash coverage. In contrast, the stochastic spatial version of the model switches between a spatially homogeneous grassland regime with high ash coverage, mirroring the steady state predicted by the mean-field GBA model, and a new grass-dominated regime with occasional fire-spreading events well separated in time (Fig. (9)) not present in the mean-field model and not previously observed in comparable spatial stochastic models of forest-savanna ecosystems (Hébert-Dufresne et al. 2018; Wuyts et al. 2017). We hypothesize that these new transient dynamics are a product of the system’s high dimensionality and the ability of the spatial extent to block large fires until the grass layer has become sufficiently dense; these phenomena are beyond the scope of the mean-field model and hence require a separate, dedicated analysis to fully quantify.

**Fig. 15:**
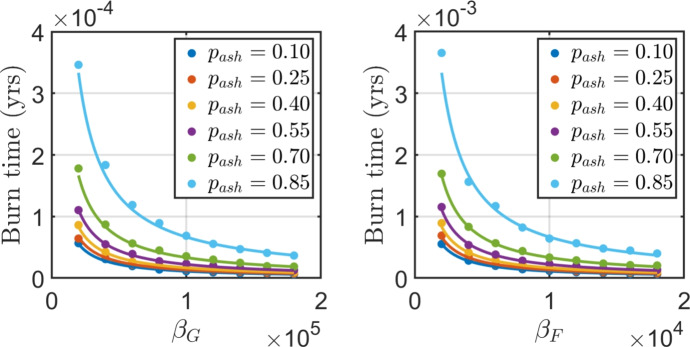
Testing the spatial transition of grass burning (left) and forest burning (right) with N = 2000. Dots show the average of 600 model simulations, and solid lines show theoretical expectations, with good agreement observed in all cases tested

Simulations of our spatial FGBA model explicitly demonstrate the hypothesized mechanisms underpinning bistability in forest tree cover. In particular, occasional ignitions followed by rapid fire spreading can maintain low forest cover even at high forest spreading rates. Meanwhile, the inability of fire to spread in regions of dense tree cover maintains high forest cover even at high fire ignition and spread rates (Fig. (12)), consistent with other recent spatial stochastic models of forest-savanna ecosystems (Hébert-Dufresne et al. 2018; Wuyts et al. 2017). In further simulations (see Appendix D.2 and Fig. (15)), the stability of the forest as a function of the parameter values showed qualitative agreement in both the mean-field and spatial models. In particular, increases in parameters such as $\varphi$ (the rate of fire spread over grass or ash) allow the system to have a finite probability of reaching a high forest state.

Our proposed FGBA model is highly versatile and can be used to study a wide range of possible forest and grassland setups. For example, differences in soil quality could be modeled by choosing vegetation sites within $\Omega$ according to a non-uniform probability distribution. One could also model forest spread via heavy-tailed, non-Gaussian spreading kernels, which may be a more accurate model of forest spread than our current assumption that forest trees only spread locally (Nathan et al. 2012). One could also investigate the impact of non-spatially uniform forest distributions, for example, if the forest were distributed into distinct regions of high tree density separated by areas of low tree density or into shapes with varying perimeter-area ratios. An essential addition to consider in future work and developments of this framework will be rainfall seasonality and its associated consequences for fire ignition. Forest-savanna ecosystems are subject to yearly wet and dry seasons that significantly impact tree growth and the relative humidity of the ecosystem, hence making the system more or less fire-prone at certain times of the year (Accatino and De Michele 2013). This will likely alter the dynamics presented in Section 4, but we believe the present analysis provides a crucial baseline against which to benchmark results that incorporate seasonality, isolating the new effects introduced by seasonal forcing.

Our model aims to alleviate several limitations of similar mathematical models of forest-savanna ecosystems, while retaining a reasonable degree of mathematical tractability. In particular, our model is spatially explicit, models dynamics on all time scales (fire, grass, and forest), allows an arbitrary distribution of vegetation sites, and incorporates arbitrary spreading kernels, enabling the study of non-isotropic or long-distance spreading. However, to avoid excessive mathematical and computational complexity, our model has some limitations. For instance, our model considers only fire and vegetation spread as transition processes in this paper, although our overarching framework can incorporate other processes. Other models consider additional factors, such as herbivory, soil layer quality, and competition between species (Holdo et al. 2022; Abades et al. 2014), which could be added to our mathematical framework at the cost of additional complexity. Moreover, we could make our model more realistic by expanding the state-space to include different savanna tree types at different life stages, as in other related models (Touboul et al. 2018). The key role of transient dynamics and stochasticity will likely remain in such extensions. Still, it would undoubtedly be important to extend the present work to these more realistic settings to relate more closely to empirical observations and hence further our understanding of these precious and endangered ecosystems.

## Figures and Tables

**Fig. 13:**
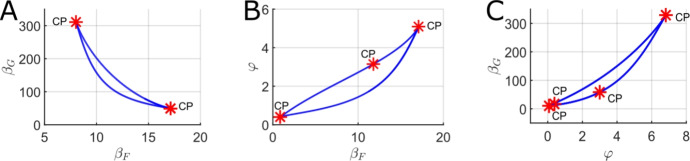
Two parameter bifurcation diagrams of the limit points in Fig. (5) for partially timescale separated parameter values. CP indicates a codimension two cusp point where two curves of saddle-node bifurcations intersect. Parameter values (fixed if not the bifurcation parameters): $\beta _G = 50$, $\beta _F = 10$, $q = 30$, $\gamma = 10$, $\varphi = \varphi _A = \varphi _G = 1$, $\mu = 0.01$, $f_0 = g_0 = 0.01$, $f_1 = 0.5$, and $g_1 = 1$

**Fig. 14:**
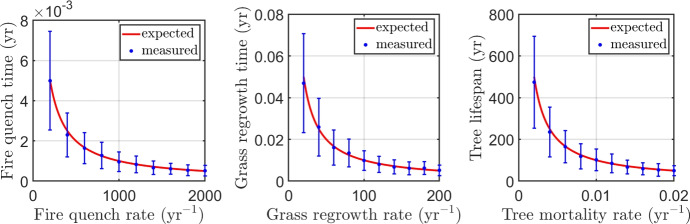
Testing the fire quench (left), grass regrowth (center), and tree mortality (right) transitions

**Fig. 16:**
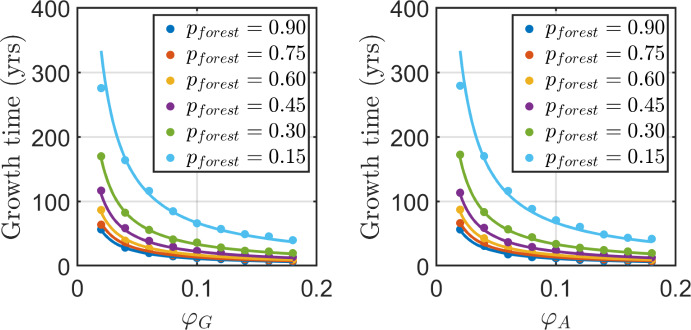
Testing of the spatial transition of forest spread into grass (left) and ash (right)

## Data Availability

The codes used in this paper are available at https://github.com/patterd2/vegetation_fire_models.

## Appendix A Mathematical Proofs

### Proof of Proposition 1 (forward invariance of the GBA subspace)

Equivalently $(G(t), B(T))$ remains contained in the closed triangle $\mathcal {T}_{GBA} = \{ (G,B) \in \mathbb {R}^2: G \ge 0, B \ge 0, G + B \le 1\}$ at all times. This is easily shown by observing that the vector field $(\dot{G}, \dot{B})$ points towards the interior of $\mathcal {T}_1$ everywhere on $\partial \mathcal {T}_1$:

$$\begin{aligned} {\left\{ \begin{array}{ll} G = 0 & \Rightarrow \dot{G} = \gamma (1- B) \ge 0\\ B = 0 & \Rightarrow \dot{B} = \Phi _G(G)G \ge 0\\ G + B = 1 & \Rightarrow \dot{G} + \dot{B} = -qB \le 0. \end{array}\right. } \end{aligned}$$

$$\square $$

### Proof of Proposition 5 (forward invariance for the nonspatial FGBA model)

Analogously to the GBA system, we check for flow invariance of the tetrahedron $\mathcal {T}_{FGBA} = \{(F,G,B) \, | \, F \ge 0, G \ge 0, B \ge 0, F +G+B \le 1\}$ to ensure that any trajectory starting at an ecologically relevant condition remains ecologically relevant at all times.

$$\begin{aligned} {\left\{ \begin{array}{ll} F = 0 & \Rightarrow \dot{F} = 0\\ G = 0 & \Rightarrow \dot{G} = \gamma (1-F-B)+\mu F \ge 0 \\ B = 0 & \Rightarrow \dot{B} = \Phi _G(G)G + \Phi _F(G)F \ge 0 \\ F +G+B = 1 & \Rightarrow \dot{F} +\dot{G} + \dot{B} = -qB \le 0, \end{array}\right. } \end{aligned}$$

so $\mathcal {T}_{FGBA}$ is invariant, as desired. $\square$

## Appendix B Computation of equilibrium burning cover value

We now examine the function $\overline{B}(F)$, which generates an equilibrium burning cover value for any input forest value (including $F=0$). To find $\overline{B}(F)$, set the four equations in system (3) to 0, forming an essentially three-dimensional system. From the equation for $\dot{A}$ we obtain

$$\begin{aligned} \overline{G}(F,B)&= 1 - F - \Big ( \frac{\gamma + \varphi F + q}{\gamma + \varphi F}\Big ) B. \end{aligned}$$

Plugging this into the equation for $\dot{B}$ then gives

$$\begin{aligned} 0&= c_0(F) + c_1(F) B + c_2(F) B^2 \end{aligned}$$

where

$$\begin{aligned} c_0(F)&= g_0(1-F) + f_0F \\ c_1(F)&= \beta _G(1-F) - g_0 \Big ( \frac{\gamma + \varphi F + q}{\gamma + \varphi F} \Big ) + \beta _F F - q \\ c_2(F)&= -\beta _G \Big ( \frac{\gamma + \varphi F + q}{\gamma + \varphi F} \Big ) \end{aligned}$$

where we work in the assumption that $F \approx 0$. A simple application of the quadratic formula then gives

$$\begin{aligned} \overline{B}(F)&= \frac{-c_1(F) \pm \sqrt{c_1^2(F) - 4c_0(F)c_2(F)}}{2c_2(F)}. \end{aligned}$$

Next note that as $g_0 \rightarrow 0$ then

$$\begin{aligned} \overline{B}(0)&= \frac{(\beta _G - q) \mp (\beta _G -q)}{2 \beta _G(\frac{\gamma + q}{\gamma })} \end{aligned}$$

where we assume that $\beta _G > q$. Comparing this expression to the GBA steady state with $g_0 \rightarrow 0$ in Eq. (8) then indicates that we must choose the + sign. Next, expand $\overline{B}(F)$ in powers of *F*:

$$\begin{aligned} \overline{B}(F)&= d_0 + d_1 F + d_2 F^3 + \mathcal {O}(F^3) \end{aligned}$$

where

$$\begin{aligned} d_0&= \frac{(\beta _G - q) \gamma }{\beta _G(q+\gamma )} + \Big (\frac{q}{\beta _G(\beta _G-q)} \Big )g_0 + \mathcal {O}(g_0^2) \\ d_1&= \frac{(\beta _f - \beta _G)\gamma (q + \gamma ) + q(\beta _G-q)\varphi }{\beta _G(q+\gamma )^2} + \Big (\frac{1}{\beta _g-q} \Big )f_0 + \Big (\frac{(q-\beta _F)(\beta _F - \beta _G)}{(q-\beta _G)^3} \Big )g_0 \\&\quad \quad \quad \quad \quad + \mathcal {O}(g_0^2, g_0 f_0, f_0^2) \\ d_2&= \frac{q\varphi ((\beta _F-\beta _G)(q+\varphi ) + (q-\beta _G)\varphi )}{\beta _G(q+\gamma )^3} + \mathcal {O}(g_0, f_0). \end{aligned}$$

Using our orders of magnitude approximation $\beta _G \approx q \gg \gamma \gg \varphi \approx 1$ and $\beta _G \gg \beta _F$ we find that

$$\begin{aligned} d_0&\approx \frac{\gamma }{q} + \frac{1}{q} g_0, \quad d_1 \approx \frac{\gamma }{q} + \frac{1}{q} (f_0 + g_0), \quad d_2 \approx \frac{\varphi }{q}, \end{aligned}$$

so $d_0 \approx d_1 \gg d_2$, and we can reasonably approximate

$$\begin{aligned} \overline{B}(F) \approx d_0 + d_1 F, \end{aligned}$$

and it is also apparent that

$$\begin{aligned} \overline{B}(0) = d_0, \quad \overline{B}'(0) = d_1. \end{aligned}$$

We can perform a similar analysis for $F \approx 1$. In this case, we write

$$\begin{aligned} \overline{B}(F) = \tilde{d}_0 + \tilde{d}_1(F - 1) + \mathcal {O}((F-1)^2) \end{aligned}$$

where

$$\begin{aligned} \tilde{d}_0&= \frac{f_0}{q-\beta _F}+ \mathcal {O}(g_1^2, g_1f_1, f_1^2), \\ \tilde{d}_1&= \Big ( \frac{q-\beta _G}{(q-\beta _F)^2} \Big ) f_1 + \Big (\frac{1}{\beta _f - q}\Big ) g_1 + \mathcal {O}(g_1^2, g_1f_1, f_1^2). \end{aligned}$$

## Appendix C Additional Numerical Bifurcation Analysis

Fig. (13) shows a number of two-parameter bifurcation diagrams in which we trace the saddle-node bifurcations from Fig. (5). The regions enclosed by the blue saddle-node curves indicate the parameter regimes in which we can expect bistability in forest cover; the intersection of two different saddle-node bifurcation curves is a codimension two cusp bifurcation point (CP), indicated with a red star (Fig. 13).

## Appendix D Simulation Testing

Below, we perform some sense checks on the model to ensure that the simulation codes are bug-free and behaves as expected. We test each transition rate separately by introducing artificial situations with readily predictable behavior and comparing the simulation results with analytic predictions.

### D.1 Testing non-spatial transitions

The simplest transitions to test are the spontaneous, non-spatial transitions governed by the parameters *q* ($B \rightarrow A$), $\gamma$ ($A \rightarrow G)$ and $\mu$ ($F \rightarrow G$) corresponding to fire quenching, grass regrowth, and tree mortality, respectively. All testing was performed with $L = 100$ and $N = 500$.

To test the fire quench rate we began the simulation with all states in the burning state and removed grass regrowth by setting $\gamma = 0$. The times at which each burning state transitioned into the ash state were recorded at various values of *q* (see Fig. 14). The quench times are expected to follow a Poisson distribution with rate *q*, giving an expected quench time of $\frac{1}{q}$ with variance $\frac{1}{q}$.

Similarly, to check the grass regrowth rate from ash we began with all sites in the ash state. We set $g_0 = g_1 = 0$ to remove spontaneous grass fires. The times at which each ash site transitioned into a grass state was recorded for various values of $\gamma$. As expected, he regrowth times followed a Poisson distribution with rate $\gamma$ and expected regrowth time $\frac{1}{\gamma }$ with variance $\frac{1}{\gamma }$.

Lastly, to check forest mortality transitions we began the simulation with all sites in the forest state and removed forest spread by setting $\varphi _G = \varphi _A = 0$. Grass and forest fires were also eliminated by setting $g_0 = g_1 = f_0 = f_1 = 0$. The times at which each forest site transitioned into a grass state was recorded at various values of $\mu$. The tree lifetimes are expected to follow a Poisson distribution wtih rate $\mu$ giving an expected lifetime of $\frac{1}{\mu }$ with variance $\frac{1}{\mu }$.

### D.2 Testing spatial transitions

We next tested the four directly spatial transitions in the FGBA model: the spread of forest through grass and through ash and the spread of fire through grass and through forest.

Fire spread through grass was tested introducing the initial condition where a single randomly chosen vegetation site in the domain would be in a grass state while the remaining sites are all randomly assigned to be either burning or ash states according to some fixed probability $p_{\text {ash}}$ of the state being ash. Further, $g_0 = g_1 = \gamma = q = 0$ was set to remove spontaneous grass ignitions and grass regrowth and prevent fire quenching. The burn rate at the grass site is then expected to be approximately $\beta _G p_{\text {ash}}$. The time at which the grass site transitioned to a burning state was then measured at various values of $\beta _G$ and $p_{\text {ash}}$. The average of 600 trials for each pair of values $(\beta _G, p_{\text {ash}})$ is plotted in Fig. (15).

Fire spread through forest was tested in an analogous situation where instead of a single grass site, a single forest site was used and $f_0 = f_1 = \mu = 0$ was set to eliminate all possible transitions except the forest to burning transition. The burn rate at the forest site is then expected to be approximately $\beta _F (1-p_{\text {ash}})$. The time at which the grass site transitioned to a burning state was then measured at various values of $\beta _F$ and $p_{\text {ash}}$. The average of 600 trials for each pair of values $(\beta _F, p_{\text {ash}})$ is plotted in Fig. (15).

Next, the spread of forest through grass was tested by randomly choosing a single vegetation site to be grass; the remaining sites are randomly chosen to be in the forest state with probability $p_{\text {forest}}$ and are otherwise in the ash state. To prevent all other possible transitions besides the grass to forest transition we set $g_0 = g_1 = f_0 = f_1 = q = \gamma = \mu = \varphi _A = 0$. Then the rate at which the grass site should transition to forest is approximately $\varphi _G p_{\text {forest}}$. The time at which the grass site transitioned to a forest state was then measured at various values of $\varphi _G$ and $p_{\text {forest}}$. The average of five trials for each pair of values $(\varphi _G, p_{\text {forest}})$ is plotted in Fig. (16).

Lastly, the spread of forest through ash was tested by randomly choosing a single vegetation site to be ash while the remaining sites are randomly chosen to be in the forest state with probability $p_{\text {forest}}$ and are otherwise in the grass state. To prevent all other possible transitions besides the ash to forest transition, we set $g_0 = g_1 = f_0 = f_1 = q = \gamma = \mu = \varphi _G = 0$. Then the rate at which the ash site should transition to forest is approximately $\varphi _A p_{\text {forest}}$. The time at which the grass site transitioned to a forest state was then measured at various values of $\varphi _A$ and $p_{\text {forest}}$. The average of five trials for each pair of values $(\varphi _A, p_{\text {forest}})$ is plotted in Fig. (16).

## Appendix E Computation of linear regimes in non-fire forest mortality bifurcation plot

Note the largely linear regimes within the non-fire forest mortality ($\mu$) bifurcation plots aside from an elbow at $F \approx 0.6$. To determine exact expressions for these two regimes, we note that the equation for $\dot{F}$ in system (3) can only vanish for $F>0$ if

$$\begin{aligned} \varphi (1-F) - (\varphi + \beta _F)(\overline{B}(F))- \Phi _F(F) - \mu = 0 \end{aligned}$$

For $F < 0.6$ we approximate $\overline{B}(F)$ as a linear function of *F* (see App. B) so that we obtain a line with x-intercept

$$\begin{aligned} \mu = \varphi - (\varphi + \beta _F)\Big ( \frac{(\beta _G-q) \gamma }{\beta _G(q+\gamma )}\Big ) - f_0 \end{aligned}$$

and slope

$$\begin{aligned} m = \frac{\beta _G(q + \gamma )^2}{\beta _F(\beta _F-\beta _G)\gamma (q + \gamma ) + q \beta _F(\beta _G-q)\varphi }. \end{aligned}$$

Similarly, for $F > 0.6$ an analogous calculation gives a line with a *y*-intercept at

$$\begin{aligned} F&= \frac{\varphi - (\varphi + \beta _F)(\tilde{d}_0 - \tilde{d}_1) - f_1}{\varphi + (\varphi + \beta _F)\tilde{d}_1} \end{aligned}$$

and slope

$$\begin{aligned} m = \frac{1}{-\varphi - (\varphi + \beta _F)\tilde{d}_1}. \end{aligned}$$

To understand the origin of the elbow at $F \approx 0.6$ we note that $\overline{B}'(F)$ is undefined when the discriminant $c_1(F)^2 - 4c_0(F)c_2(F)$ vanishes. To zeroth order in $f_0$ and $g_0$ we then find that $\overline{B}'(F)$ is undefined when

$$\begin{aligned} F^*&= \frac{q - \beta _G}{\beta _G - \beta _F}. \end{aligned}$$

Furthermore, to zeroth order in $f_0$ and $g_0$

$$\begin{aligned} \overline{B}'(F) = {\left\{ \begin{array}{ll} 0 & F > F^* \\ \frac{-q^2\varphi + q \beta _G(-\gamma + \varphi - 2F \varphi )+(\beta _F-\beta _G)(\gamma + F \varphi )^2 + q\beta _F(\gamma + 2 \varphi )}{\beta _G(q + \gamma + F \varphi )^2} & F < F^*. \end{array}\right. } \end{aligned}$$

Note that all *F*-dependent terms are suppressed since $q \gg \gamma \gg \varphi$ so $\overline{B}'(F)$ is approximately constant in its two regimes, so $\overline{B}(F)$ is approximately linear.
